# Comparative transcriptomics analysis reveals difference of key gene expression between banana and plantain in response to cold stress

**DOI:** 10.1186/s12864-015-1551-z

**Published:** 2015-06-10

**Authors:** Qiao-Song Yang, Jie Gao, Wei-Di He, Tong-Xin Dou, Li-Jie Ding, Jun-Hua Wu, Chun-Yu Li, Xin-Xiang Peng, Sheng Zhang, Gan-Jun Yi

**Affiliations:** Institute of Fruit Tree Research, Guangdong Academy of Agricultural Sciences, 80 Dafeng 2nd street, Tianhe District, Guangzhou, Guangdong Province 510640 China; Key Laboratory of South Subtropical Fruit Biology and Genetic Resource Utilization, Ministry of Agriculture, Guangzhou, 510640 China; State Key Laboratory for Conservation and Utilization of Subtropical Agro-bioresources, South China Agricultural University, Guangzhou, 510640 China; National Key Laboratory of Crop Genetic Improvement, Huazhong Agricultural University, Wuhan, 430070 China; Institute of Biotechnology, Cornell University, Ithaca, NY 14853-2703 USA

**Keywords:** Comparative transcriptome analysis, Cold tolerance, Banana, Plantain, ICE1, MYBS3 pathways

## Abstract

**Background:**

Banana and plantain (*Musa spp.*) comprise an important part of diets for millions of people around the globe. Low temperature is one of the key environmental stresses which greatly affects the global banana production. To understand the molecular mechanism of the cold-tolerance in plantain we used RNA-Seq based comparative transcriptomics analyses for both cold-sensitive banana and cold-tolerant plantain subjected to the cold stress for 0, 3 and 6 h.

**Results:**

The cold-response genes at early stage are identified and grouped in both species by GO analysis. The results show that 10 and 68 differentially expressed genes (DEGs) are identified for 3 and 6 h of cold stress respectively in plantain, while 40 and 238 DEGs are identified respectively in banana. GO classification analyses show that the majority of DEGs identified in both banana and plantain belong to 11 categories including regulation of transcription, response to stress signal transduction, etc. A similar profile for 28 DEGs was found in both banana and plantain for 6 h of cold stress, suggesting both share some common adaptation processes in response to cold stress. There are 17 DEGs found uniquely in cold-tolerance plantain, which were involved in signal transduction, abiotic stress, copper ion equilibrium, photosynthesis and photorespiration, sugar stimulation, protein modifications etc. Twelve early responsive genes including *ICE1* and *MYBS3* were selected and further assessed and confirmed by qPCR in the extended time course experiments (0, 3, 6, 24 and 48 h), which revealed significant expression difference of key genes in response to cold stress, especially *ICE1* and *MYBS3* between cold-sensitive banana and cold-tolerant plantain.

**Conclusions:**

We found that the cold-tolerance pathway appears selectively activated by regulation of *ICE1* and *MYBS3* expression in plantain under different stages of cold stress*.* We conclude that the rapid activation and selective induction of ICE1 and MYBS3 cold tolerance pathways in plantain*,* along with expression of other cold-specific genes, may be one of the main reasons that plantain has higher cold resistance than banana*.*

**Electronic supplementary material:**

The online version of this article (doi:10.1186/s12864-015-1551-z) contains supplementary material, which is available to authorized users.

## Background

Low temperature is one of the key environmental stresses that many plants have to cope with during their life cycle, which can influence growth, development, as well as the yield, quality, postharvest life, and geographic distribution of crop plants [1,2]. Cold stress can be classified as chilling (0-15°C) and freezing (<0°C) stresses. Generally, plants from temperate regions have the capacity to cold acclimate, that is, to develop increased freezing tolerance after being exposed to low, nonfreezing temperatures [3], but many important crops, such as rice, maize, soybean, cotton, and tomato, which originated in the tropics and subtropics, lack the cold acclimation mechanism and are sensitive to chilling stress [4]. Moreover, different varieties of the same species can also exhibit a high degree of genetic variability for cold tolerance [5-7]. In conventional crop cross-breeding, the cold-tolerant varieties are usually used as the main resource for increasing the cold tolerance of cultivars [8]. However, the lack of detailed knowledge of molecular mechanisms responsible for cold stress limits the potential for crop improvement. Investigation of gene expression profiles in response to cold stress will shed light on the sensing and regulatory networks for cold acclimation in plants and provide an effective approach to select targeted candidate genes for manipulation and/or cross-breeding of agronomic plants [8].

*Musa spp*. (including Banana and Plantain), which originated in the tropics are giant perennial herbaceous monocots. They are vital staple food in many African countries and the most popular fruit in industrialized countries [9]. *Musa spp*. exhibits a high degree of genetic variability for cold tolerance, with banana (*Musa spp.* Cavendish; AAA Group) being more cold sensitive than plantain (*Musa spp.* Dajiao; ABB Group). When the temperature drops to 8°C, banana growth is arrested, injury is inflicted [10], and irreversible damage often occurs with temperatures below 12°C [11]. In milder cases of cold injury, the fruit can be harvested, but its ripening process is abnormal. In contrast to the Cavendish “dessert” banana, the plantain species, Dajiao has superior cold tolerance, enabling it to tolerate temperatures of 0-4°C [12], and has been proposed as a potential germplasm resource of cold tolerance in banana breeding [10].

Although *Musa spp*. appear to lack a cold acclimation mechanism, transgenic plantain constitutively overexpressing the *Arabidopsis* transcription factor *DREB1B/CBF1* becomes highly tolerant to cold by increasing SOD activities, decreasing MDA content and the electrolyte leakage rate. Meanwhile the growth rates of these transgenic plants are severely retarded under normal growth conditions [13]. In the herbaceous monocot rice (*Oryza sativa*), which also originated in the tropics, a novel MYBS3-dependent pathway has recently been identified as essential for cold tolerance. MYBS3 was found to repress the CBF-dependent cold signaling pathway. Molecular evidence indicates that the sequential expression of *CBF* and *MYBS3* provides two complementary mechanisms for conferring cold tolerance, with the *CBF*-mediated process initiating the immediate cold shock response and the *MYBS3*-mediated system adjusting the long-term cold adaptation in rice [14]. Recently, a new quantitative trait locus *COLD1* was identified, which interacts with G protein to activate the Ca^2+^ channel for low temperature sensing that confers chilling tolerance in *japonica* rice, compared with cold-sensitive *indica* rice. Overexpression of *COLD1*^*jap*^ significantly enhances chilling tolerance, whereas rice lines with deficiency or down-regulation of *COLD1*^*jap*^ are sensitive to cold stress [15]. In the past decade, some physiological responses to cold stress were analyzed, comparatively, between banana and plantain [16], and another cold-resistance related plantain *MpRCI* (Rare cold-induced gene) has been identified, that enhances low temperature resistance when heterologously expressed in transgenic tobacco [17].

Through the traditional map-based cloning and transgenic methods, one can identify some key stress-related genes. But it’s generally tedious and time-consuming, especially for fruit trees which have a relatively long juvenile phase. In recent years, advances in novel high-throughput sequencing technologies, such as Solexa/Illumina RNA-Seq (RNA sequencing) and digital gene expression (DGE) has provided an opportunity to explore cold resistance and signaling-associated genes in different species by *denovo* assembly or mapping, facilitating rapid identification and analysis of many transcriptomes [18]. Transcriptome research has become an effective means to investigate the gene expression patterns of fruit trees in the growth and development process and under various stresses, which has been reported in tropical and subtropical fruit trees, such as litchi [19,20], mango [21], papaya [22], citrus [23] and grape [24] etc.

Our quantitative proteomic analysis reveals that molecular mechanisms for the higher cold resistance found in plantain are associated with increased redox potential characterized by adapted ROS scavenging capability, reduced ROS production, decreased lipid peroxidation, and cell wall stabilization [10]. Gene expression under cold stress is very sensitive and it depends on the species, the temperature and the length of exposure to low temperature [5-7]. Although several important clues are suggested from intensive proteomic research of cold-tolerance plantain and comparing those results with functional gene analysis conducted in other plants, a comprehensive differential transcriptome analysis in response to cold stress between banana and plantain has not been reported and remains unknown.

In this study, plantain collected from a subtropical region of China with high cold-tolerance was used to investigate responses to cold stress at the transcriptional level. A cold-sensitive species, banana was examined as a control. A well-established whole genome transcriptome analysis method, based on RNA-Seq, and incorporating real time-PCR, was utilized to screen the differential transcripts associated with cold tolerance between banana and plantain. Our study provides a broad picture of the banana and plantain cold-responsive transcriptomes, with a new insight to cold-tolerance molecular mechanisms of plantain under cold stress.

## Results

### Phenotypes and electrolyte leakage of banana and plantain to cold stress

**Figure 1:**
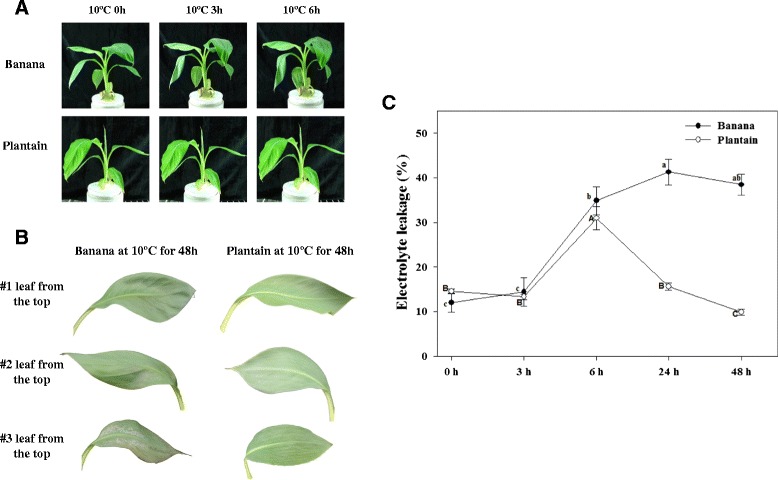
Phenotypic and physiological responses of banana and plantain under cold stress. Six-leaf stage seedlings of banana and plantain were treated at 10°C for 0, 3 and 6 h **(A)**; and comparison of phenotypic difference of the three leaves from the top of banana and plantain following 48 h of cold treatment **(B)**; the relative electrolyte leakage was determined for the banana and plantain treated at 10°C for 0, 3, 6, 24 and 48 h. The different letters (lowercase letters-banana, capital letters-plantain) labeled above columns indicate a significant difference at p ≤ 0.05 between the columns by Duncan’s test using SPSS statistical software (version 16.0, SPSS Inc. Chicago, IL). The columns with the same letters mean no significant difference (p > 0.05) between each other **(C)**.

To accurately evaluate the tolerance of plantain, we compared the physiological changes of plantain versus banana in parallel during cold stress. A significant phenotypic difference is that the banana leaves drooped after 6 h of cold treatment and the second and third leaves from the top displayed severe necrosis and wilting symptoms after 48 h of cold treatment at 10°C, while plantain leaves remained normal (Figure 1A and B). The relative leakage increased from 12.0 to 33.9% in banana leaves, and from 14.6 to 28.0% in plantain leaves respectively after 6 h of cold treatment. After 48 h of cold stress, the leakage of plantain leaves decreased to the basal level, but the leakage of banana leaves still increased and the damage was not alleviated (Figure 1C). These observations suggest that the seedlings of plantain leaves had adapted to the cold stress to some extent after 6 h of treatment and appeared to protect the membrane from further damage, suggesting that some important cold-tolerant genes, such as the early signal transduction genes of plantain, may work at the beginning 6 h of cold treatment. Both phenotypic response and relative leakage of plantain support the notion that plantain once again, has stronger cold tolerance than banana.

### RNA-Seq and alignment of unique reads to banana reference genome

The raw data quality assessment of banana and plantain libraries with control and cold treatment are shown in Additional file 1: Table S1. For banana, after filtering out low quality reads, raw reads containing ‘N’ and adaptor sequences, 12.66 M to 16.96 M clean reads were sequenced from four biological replicates of 0, 3 and 6 h cold treated libraries, respectively (Table 1). Clean reads were aligned to banana reference genome database (doubled haploid *Musa. acuminata* genotype, A genome, http://banana-genome.cirad.fr/content/download-dh-pahang). About 60% Cavendish clean reads mapped to the reference genome. Multiple mapped clean reads in each library were excluded from further analysis. Finally a total of 5.14 M to 7.55 M uniquely mapped clean reads were used for subsequent analysis. The distribution of unique reads with chromosome ‘+/−’ chain and splice/non-splice in each library were counted and are shown in Table 1.

**Table 1:** **Statistic analysis of Cavendish and Dajiao reads mapped to** ***M. acuminate*** **reference genome**

**Sample name**	**Cavendish (** ***Musa spp.*** **Cavendish; AAA group)**			**Dajiao (** ***Musa spp.*** **Dajiao; ABB group)**		
	**10°C**	**10°C**	**10°C**	**10°C**	**10°C**	**10°C**
	**0 h replicates**	**3 h replicates**	**6 h replicates**	**0 h replicates**	**3 h replicates**	**6 h replicates**
**Total reads** ^***a***^	16.96 ± 0.93	14.54 ± 0.03	12.66 ± 1.41	13.94 ± 1.51	15.03 ± 2.03	16.20 ± 0.33
**Total mapped**	10.30 ± 0.78	8.57 ± 0.18	7.59 ± 0.79	8.62 ± 0.93	9.27 ± 1.16	9.10 ± 0.13
	(60.70 ± 1.24%)	(58.94 ± 1.34%)	(59.98 ± 0.47%)	(61.86 ± 0.01%)	(61.75 ± 0.59%)	(56.20 ± 0.31%)
**Multiple mapped**	2.75 ± 0.06	3.12 ± 0.37	2.45 ± 0.09	3.20 ± 0.32	4.23 ± 0.82	3.17 ± 1.19
	(16.22 ± 0.52%)	(21.44 ± 2.50%)	(19.46 ± 1.50%)	(23.31 ± 4.79%)	(28.02 ± 1.65%)	(19.67 ± 7.75%)
**Uniquely mapped**	7.55 ± 0.71	5.45 ± 0.55	5.14 ± 0.70	5.43 ± 1.25	5.04 ± 0.35	5.94 ± 1.32
	(44.48 ± 1.76%)	(37.51 ± 3.84%)	(40.53 ± 1.02%)	(38.55 ± 4.80%)	(33.74 ± 2.23%)	(36.54 ± 7.44%)
**Reads map to ‘+’**	1.83 ± 0.22	1.33 ± 0.11	1.18 ± 0.07	1.25 ± 0.18	1.24 ± 0.10	1.46 ± 0.20
	(10.75 ± 0.70%)	(9.17 ± 0.76%)	(9.34 ± 0.52%)	(8.94 ± 0.32%)	(8.33 ± 0.46%)	(9.02 ± 1.04%)
**Reads map to ‘-’**	5.73 ± 0.49	4.12 ± 0.44	3.97 ± 0.64	4.18 ± 1.07	3.79 ± 0.25	4.47 ± 1.12
	(33.7 ± 1.06%)	(28.34 ± 3.08%)	(31.19 ± 1.54%)	(29.61 ± 4.47%)	(25.41 ± 1.78%)	(27.52 ± 6.40%)
**Non-splice reads**	7.10 ± 0.64	5.16 ± 0.52	4.92 ± 0.67	5.19 ± 1.17	4.83 ± 0.32	5.62 ± 1.13
	(41.79 ± 1.50%)	(35.51 ± 3.65%)	(38.79 ± 0.99%)	(36.86 ± 4.41%)	(32.37 ± 2.23%)	(34.59 ± 6.30%)
**Splice reads**	0.46 ± 0.07	0.29 ± 0.027	0.22 ± 0.029	0.24 ± 0.08	0.21 ± 0.03	0.32 ± 0.19
	(2.69 ± 0.27%)	(2.00 ± 0.18%)	(1.75 ± 0.029%)	(1.69 ± 0.39%)	(1.38 ± 0.00%)	(1.95 ± 1.13%)

^*a*^Total reads: clean data obtained after filtering the reads containing adapter, poly-N and low quality reads from raw data.

(Unit: Million reads).

**Table 2:** **Differentially expressed genes (DEGs) in Cavendish and Dajiao during cold treatment for 3 and 6 hours**

		**Cavendish**	**Unique in Cavendish**	**Dajiao**	**Unique in Dajiao**	**Common in both**
DEGs at 3 h	Total	40	33	10	3	7
	Up-regulated	33	26	9	2	7
	Down-regulated	7	7	1	1	0
DEGs at 6 h	Total	238	188	68	18	50
	Up-regulated	195	149	54	8	46
	Down-regulated	43	39	14	10	4

For plantain, after filtering out low quality reads, raw reads containing ‘N’ and adaptor sequences, 13.94 M to 16.20 M clean reads were generated from four biological replicates of 0, 3 and 6 h cold treated libraries, respectively (Table 1). Clean reads were aligning to banana reference genome database. There are also about 60% of plantain clean reads mapped to the reference genome. Finally a total of 5.04 M to 5.94 M uniquely mapped clean reads were used for further analysis. The distribution of unique reads with chromosome ‘+/−’ chain and splice/non-splice in each library were counted and are shown in Table 1.

### Differential expression profiling of cold stress between banana and plantain

**Figure 2:**
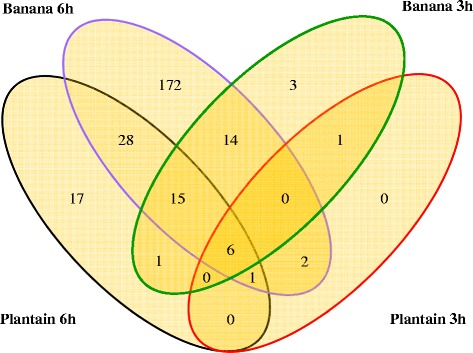
Venn diagram of differentially expressed genes (DEGs) identified for cold-sensitive banana and cold-tolerant plantain in response to cold stress. Green circle segment: the number of DEGs in banana under cold treatment at 10°C for 3 h with 0 h of banana as a control; Purple circle segment: the number of DEGs in banana under cold treatment at 10°C for 6 h, 0 h of banana as a control; Red circle segment: the number of DEGs in plantain under cold treatment at 10°C for 3 h, 0 h of plantain as a control; Black circle segment: the number of DEGs in plantain under cold treatment at 10°C for 6 h, 0 h of plantain as a control.

To comprehensively investigate the differences in gene expression between cold-sensitive banana and cold-tolerant plantain in response to cold stress, we performed comparative transcriptome analysis using the aligned reads (above). After 3 h of cold stress, a total of 40 (33 up- and 7 down-regulated), 10 (9 up- and 1 down-regulated) cold-responsive genes were identified in banana and plantain, respectively (Table 2, Figure 2, Additional file 2: Table S2 and Additional file 3: Table S3) with the threshold of 0.05 for Corrected *P*-value and 1 for log2 base of fold-change. Out of the 40 DEGs, 33 cold-responsive genes (26 up- and 7 down-regulated) were exclusively identified in banana, whereas 3 cold-responsive genes (2 up- and 1 down-regulated) were uniquely observed in plantain. The remaining 7 genes (all up-regulated) were commonly regulated by cold stress in both banana and plantain. After 6 h of cold stress, a total of 238 (195 up- and 43 down-regulated), 68 (54 up- and 14 down-regulated) cold-responsive genes were identified in banana and plantain, respectively (Table 2, Figure 2, Additional file 2: Table S2 and Additional file 3: Table S3). Out of the 238 DEGs, 188 cold-responsive genes (149 up- and 39 down-regulated) were exclusively identified in banana, whereas 18 cold-responsive genes (8 up- and 10 down-regulated) were uniquely observed in plantain and 50 genes (46 up- and 4 down-regulated) were commonly regulated by cold stress in both banana and plantain. The small number of cold-responsive genes identified in cold-tolerant plantain suggests that some inherent adaptation and regulation mechanisms may be attributed to the cold tolerance in plantain.

The DEGs with at least a 2-fold change are shown in Additional file 4: Table S4 and Table 3. After 3 h of cold stress, 7 up-regulated genes commonly identified in both banana and plantain, include GSMUA_Achr9G07610_001 (Probable cytosolic iron-sulfur protein assembly protein 1), GSMUA_Achr6G32910_001 (Zinc finger CCCH domain-containing protein 33), GSMUA_ Achr2G13410_001 (Zinc finger protein 1), GSMUA_Achr6G25670_001 (U-box domain- containing protein 25), GSMUA_Achr7G26580_001(UDP-glucose 6-dehydrogenase), GSMUA_Achr7G05900_001 (Dehydration-responsive element-binding protein 1D) and GSMUA_Achr3G23000_001 (Calcium-binding protein KIC). Meanwhile, 2 up-regulated genes including GSMUA_Achr8G21550_001 (Ethylene insensitive 3-like 1 protein) and GSMUA_Achr3G05220_001 (Probable xyloglucan endotransglucosylase/ hydrolase protein 23) and 1 down-regulated gene: GSMUA_Achr3G04360_001 (Ethylene-responsive transcription factor RAP2-13) were significantly changed in plantain at 3 hours. Interestingly, the above 3 genes with the identical trend of changes were present in banana after 6 h of cold stress, suggesting that those 3 genes be specifically responsive to cold stress, and their early response maybe critical in the cold resistance. In addition, this timely delay of response to cold stress was also found in plantain. Out of 33 DEGs found in banana after 3 h of cold stress, 16 genes with the identical response profiling were present in plantain only after 6 h of cold stress. Those 16 genes include GSMUA_Achr5G21050_001 (Ethylene-responsive transcription factor 1), GSMUA_Achr7G06910_001 (Ethylene-responsive transcription factor ERF105), GSMUA_Achr3G01650_001 (Ethylene responsive transcription factor RAP2-4), GSMUA_Achr9G04780_001 (CBL-interacting serine/threonine protein kinase 11), GSMUA_Achr7G21780_001(NAC domain-containing protein 68), GSMUA_Achr3G11070 _001 (Zinc finger A20 and AN1 domain-containing stress associated protein 11), GSMUA_Achr10G22580_001 (E3 ubiquitin-protein ligase RING1), GSMUA_Achr 7G21070_001 (Probable mannan synthase 9), GSMUA_Achr10G25070_001 (Mitochondrial 2-oxoglutarate/malate carrier protein), GSMUA_Achr7G00180_001 (Thiamine thiazole synthase 2), GSMUA_Achr6G13020_001 (Protein MKS1), GSMUA_Achr10G24780_001 (Unknown) with up-regulation, and GSMUA_Achr11G02890_001 (DNA directed RNA polymerase subunit alpha), GSMUA_Achr7G22100_001 (Expansin-A4) and GSMUA_Achr 8G30530_001 (Unknown) with down-regulation. These delayed, expressed genes may be of importance to the adaptation of cold stress for sustainable cold tolerance in plantain.

**Table 3:** **Primary functional classification on differentially expressed genes of Dajiao with fold changes > or < 2-fold**

**Gene_id**	**RPKM Dajiao 0 h**	**RPKM Dajiao 3 h**	**RPKM Dajiao 6 h**	**log2Fold Change 3 h**	**log2Fold Change 6 h**	**Gene name**
**Regulation of transcription**						
GSMUA_Achr8G25220_001	0.00		74.93		Inf	sp|Q9FDW1|MYB44_ARATH Transcription factor MYB44
GSMUA_Achr6G15840_001	0.00		244.92		Inf	sp|Q9SKD9|WRK46_ARATH Probable WRKY transcription factor 46
GSMUA_Achr7G21780_001	3.92		134.30		5.08	sp|Q52QH4|NAC68_ORYSJ NAC domain-containing protein 68
GSMUA_Achr5G07590_001	3.49		98.07		4.79	sp|Q52QH4|NAC68_ORYSJ NAC domain-containing protein 68
GSMUA_Achr6G32330_001	13.18		144.11		3.35	sp|Q52QH4|NAC68_ORYSJ NAC domain-containing protein 68
GSMUA_Achr7G06910_001	92.07		2467.93		4.67	sp|Q8VY90|EF105_ARATH Ethylene-responsive transcription factor ERF105
GSMUA_Achr5G21050_001	8.88		112.43		3.54	sp|Q40476|ERF1_TOBAC Ethylene-responsive transcription factor 1
GSMUA_Achr2G07300_001	89.60		623.19		2.69	sp|Q6K7E6|ERF1_ORYSJ Ethylene-responsive transcription factor 1
GSMUA_Achr8G06870_001	16.66		182.33		3.38	sp|Q9LW49|ERF4_NICSY Ethylene-responsive transcription factor 4
GSMUA_Achr4G33270_001	16.25		187.60		3.42	sp|Q9FE67|ERF80_ARATH Ethylene-responsive transcription factor 9
GSMUA_Achr3G01650_001	31.65		237.25		2.80	sp|Q8H1E4|RAP24_ARATH Ethylene-responsive transcription factor RAP2-4
GSMUA_Achr7G11200_001	23.57		226.28		3.11	sp|Q2R2W1|ADO3_ORYSJ Adagio-like protein 3
GSMUA_Achr8G21550_001	37.31	181.43	228.12	2.24	2.50	sp|Q9SLH0|EIL1_ARATH ETHYLENE INSENSITIVE 3-like 1 protein
GSMUA_Achr9G17080_001	176.04		810.19		2.09	sp|Q9SLH0|EIL1_ARATH ETHYLENE INSENSITIVE 3-like 1 protein
GSMUA_Achr6G32910_001	61.08	345.11	352.14	2.46	2.41	sp|Q688R3|C3H33_ORYSJ Zinc finger CCCH domain-containing protein 33
GSMUA_Achr11G02890_001	407.44		97.21		−2.17	sp|A6MMN9|RPOA_DIOEL DNA-directed RNA polymerase subunit alpha
GSMUA_Achr3G04360_001	195.09	7.20		−4.82		sp|Q9LM15|RA213_ARATH Ethylene-responsive transcription factor RAP2-13
**Response to stress**						
GSMUA_Achr7G05900_001	4.55	474.74	496.55	6.76	6.74	sp|Q9LWV3|DRE1D_ORYSJ Dehydration-responsive element-binding protein 1D
GSMUA_Achr6G14750_001	1.82		97.73		5.72	sp|Q9SYM2|STHY_ARATH Probable salt tolerance-like protein At1g78600
GSMUA_Achr5G07340_001	1.61		80.13		5.62	sp|Q10MX1|P2C32_ORYSJ Probable protein phosphatase 2C 32
GSMUA_Achr6G13020_001	9.58		343.69		4.98	sp|Q8LGD5|MKS1_ARATH Protein MKS1
GSMUA_Achr2G13410_001	16.68	246.83	243.05	3.87	3.76	sp|Q42430|ZFP1_WHEAT Zinc finger protein 1
GSMUA_Achr3G11070_001	208.08		1667.24		2.91	sp|Q84PD8|SAP11_ORYSJ Zinc finger A20 and AN1 domain-containing stress-associated protein 11
GSMUA_Achr10G22580_001	46.34		325.56		2.74	sp|P0CH30|RING1_GOSHI E3 ubiquitin-protein ligase RING1
GSMUA_Achr7G27650_001	234.49		1072.10		2.10	sp|P49310|GRP1_SINAL Glycine-rich RNA-binding protein GRP1A
GSMUA_Achr7G00180_001	656.47		2428.04		1.76	sp|F6H7K5|THI42_VITVI Thiamine thiazole synthase 2, chloroplastic
GSMUA_Achr8G20530_001	547.88		145.12		−2.04	sp|P11432|ELI_PEA Early light-induced protein, chloroplastic
GSMUA_Achr7G20770_001	681.33		129.38		−2.53	sp|Q9MA41|CTL1_ARATH Chitinase-like protein 1
GSMUA_Achr3G03820_001	180.03		20.24		−3.28	sp|Q00874|DR100_ARATH DNA-damage-repair/toleration protein DRT100
GSMUA_Achr1G17220_001	758.94		19.48		−5.40	sp|Q9SW93|SCA_LILLO Stigma/stylar cysteine-rich adhesin
**Protein modification**						
GSMUA_Achr9G08750_001	21.54		308.06		3.74	sp|Q9LT79|PUB25_ARATH U-box domain-containing protein 25
GSMUA_Achr6G25670_001	128.52	845.34	1563.67	2.68	3.48	sp|Q9LT79|PUB25_ARATH U-box domain-containing protein 25
GSMUA_Achr7G01430_001	52.60		429.81		2.95	sp|P05332|YP20_BACLI Uncharacterized N-acetyltransferase p20
GSMUA_Achr2G13700_001	15.09		110.79		2.79	sp|Q10469|MGAT2_HUMAN Alpha-1,6-mannosyl-glycoprotein 2-beta-N-acetylglucosaminyltransferase
GSMUA_Achr6G18960_001	455.83		2228.34		2.19	sp|P0CH33|UBQ11_ARATH Polyubiquitin 11
**Signal transduction**						
GSMUA_Achr9G04780_001	0.00		111.31		Inf	sp|O22932|CIPKB_ARATH CBL-interacting serine/threonine-protein kinase 11
GSMUA_Achr4G16550_001	13.05		123.18		3.19	sp|Q94BT2|AIR12_ARATH Auxin-induced in root cultures protein 12
GSMUA_Achr1G03980_001	58.82		544.78		3.15	sp|Q9ZPX9|KIC_ARATH Calcium-binding protein KIC
GSMUA_Achr3G23000_001	66.66	705.10		3.41		sp|Q9ZPX9|KIC_ARATH Calcium-binding protein KIC
GSMUA_Achr6G35120_001	299.39		1905.08		2.58	sp|Q9LNE7|CML7_ARATH Calmodulin-like protein 7
**Cell wall organization and biogenesis**						
GSMUA_Achr7G21070_001	12.21		113.07		3.14	sp|Q67VS7|CSLA9_ORYSJ Probable mannan synthase 9
GSMUA_Achr6G05390_001	50.30		294.18		2.46	sp|Q9LR44|U75B1_ARATH UDP-glycosyltransferase 75B1
GSMUA_Achr3G05220_001	0.00	108.92		Inf		sp|Q38910|XTH23_ARATH Probable xyloglucan endotransglucosylase/hydrolase protein 23
GSMUA_Achr3G26220_001	349.74		19.19		−4.32	sp|Q8LER3|XTH7_ARATH Probable xyloglucan endotransglucosylase/hydrolase protein 7
GSMUA_Achr7G22100_001	376.14		6.12		−6.08	sp|Q0DHB7|EXPA4_ORYSJ Expansin-A4
**Transport**						
GSMUA_Achr10G25070_001	42.74		561.50		3.66	sp|Q9CR62|M2OM_MOUSE Mitochondrial 2-oxoglutarate/malate carrier protein
GSMUA_Achr7G16570_001	66.35		290.42		2.03	sp|P46032|PTR2_ARATH Peptide transporter PTR2
GSMUA_Achr1G22700_001	334.68		1346.56		1.93	sp|Q8VZ80|PLT5_ARATH Polyol transporter 5
**Nucleosome assembly**						
GSMUA_Achr1G07160_001	64.61		491.53		2.84	sp|P0CG89|H4_SOYBN Histone H4
GSMUA_Achr7G12120_001	443.53		1635.08		1.76	sp|Q9M5W4|H1_EUPES Histone H1
Cell redox homeostasis						
GSMUA_Achr3G25160_001	0.00		53.07		Inf	sp|Q84JR9|TTL4_ARATH TPR repeat-containing thioredoxin TTL4
**Iron-sulfur cluster assembly**						
GSMUA_Achr9G07610_001	0.00	343.42	275.72	Inf	Inf	sp|Q6FJ73|CIAO1_CANGA Probable cytosolic iron-sulfur protein assembly protein 1
**Proteolysis**						
GSMUA_Achr11G04410_001	1.46		120.93		6.34	sp|Q766C3|NEP1_NEPGR Aspartic proteinase nepenthesin-1
**Oxidation reduction**						
GSMUA_Achr7G26580_001	83.33	882.49	1084.18	3.35	3.57	sp|Q96558|UGDH_SOYBN UDP-glucose 6-dehydrogenase
**RNA modification**						
GSMUA_Achr4G11450_001	61.95		416.78		2.64	sp|Q9SKZ2|CAF1G_ARATH Probable CCR4-associated factor 1 homolog 7
**Copper ion homeostasis**						
GSMUA_Achr3G16210_001	9685.89		33776.00		1.72	sp|Q40256|MT3_MUSAC Metallothionein-like protein type 3
**Photosynthesis and photorespiration**						
GSMUA_Achr10G19930_001	210.99		18.46		−3.62	sp|O24045|RBS_MUSAC Ribulose bisphosphate carboxylase small chain, chloroplastic
**Response to sucrose stimulus**						
GSMUA_Achr11G11670_001	233.57		3.50		−6.15	sp|Q9LZP9|CP122_ARATH Calvin cycle protein CP12-2, chloroplastic
**Unknown**						
GSMUA_Achr2G22400_001	0.00		59.28		Inf	Putative uncharacterized protein
GSMUA_Achr10G24780_001	2.98		136.75		5.50	Putative uncharacterized protein
GSMUA_Achr6G10300_001	12.59		230.91		4.17	Putative uncharacterized protein
GSMUA_Achr2G22290_001	28.69		317.86		3.39	Putative uncharacterized protein
GSMUA_Achr5G01760_001	40.45		432.42		3.35	Putative uncharacterized protein
GSMUA_Achr3G28910_001	263.62		1904.50		2.75	Putative uncharacterized protein
GSMUA_Achr6G16830_001	61.64		364.03		2.45	Putative uncharacterized protein
GSMUA_Achr8G05250_001	163.34		933.13		2.41	Putative uncharacterized protein
GSMUA_Achr4G24680_001	409.42		52.57		−3.08	Putative uncharacterized protein
GSMUA_Achr8G30530_001	245.64		28.47		−3.20	Putative uncharacterized protein
GSMUA_Achr6G01490_001	158.51		11.52		−3.90	Putative uncharacterized protein
GSMUA_Achr9G12290_001	65.93		0.00		-Inf	Putative uncharacterized protein
GSMUA_Achr9G26820_001	65.88		0.00		-Inf	Putative uncharacterized protein

There are 45 more DEGs being identified in plantain for 6 h of cold stress. Of which 28 genes include GSMUA_Achr8G25220_001 (Transcription factor MYB44), GSMUA_Achr 6G15840_001 (Probable WRKY transcription factor 46), GSMUA_Achr3G25160_001 (TPR repeat-containing thioredoxin TTL4), GSMUA_Achr11G04410_001 (Aspartic proteinase nepenthesin-1), GSMUA_Achr6G14750_001 (Probable salt tolerance-like protein), GSMUA_ Achr5G07340_001 (Probable protein phosphatase 2C), GSMUA_Achr4G33270_001 (Ethylene-responsive transcription factor 9), GSMUA_Achr8G06870_001 (Ethylene responsive transcription factor 4), GSMUA_Achr7G11200_001 (Adagio-like protein 3), GSMUA_Achr7G01430_001 (Uncharacterized N-acetyltransferase p20), GSMUA_Achr 2G13700_001(Alpha-1,6-mannosyl-glycoprotein2-beta-N-acetylglucosaminyl transferase), GSMUA_Achr4G11450_001 (Probable CCR4-associated factor 1 homolog 7), GSMUA_ Achr6G16830_001 (Pentatricopeptide repeat-containing protein), GSMUA_ Achr6G18960 _001 (Polyubiquitin 11), GSMUA_Achr7G27650_001 (Glycine-rich RNA binding protein GRP1A), GSMUA_Achr1G22700_001 (Polyol transporter 5), GSMUA_ Achr7G12120_001 (Histone H1), GSMUA_Achr3G03820_001(DNA-damage-repair/toleration protein DRT100), GSMUA_Achr2G22400_001 (Unknown), GSMUA_Achr6G10300_001 (Unknown), GSMUA_ Achr2G22290_001 (Unknown), GSMUA_ Achr5G01760_001 (Unknown), GSMUA_Achr 6G35120_001 (Unknown). A similar differential profile for those 28 genes was also found in banana for 6 h of cold stress, suggesting they share the similar adaptation mechanism to cold stress. The remaining 17 genes were differentially expressed only in plantain (no significant changes in banana). These genes include GSMUA_Achr4G16550_001 (Auxin-induced in root cultures protein 12), GSMUA_Achr1G07160_001 (Histone H4), GSMUA_ Achr6 G05390_001(UDP-glycosyltransferase 75B1), GSMUA_Achr7G16570_001 (Peptide transporter PTR2), GSMUA_Achr3G16210_001 (Metallothionein-like protein type 3), GSMUA_Achr3G28910_001 (Unknown), GSMUA_Achr8G05250_001 (Unknown) with up-regulation, and GSMUA_Achr8G20530_001 (Early light-induced protein), GSMUA_ Achr7G20770_001 (Chitinase-like protein 1), GSMUA_Achr4G24680_001 (21 kDa protein), GSMUA_Achr10G19930_001 (Ribulose bisphosphate carboxylase small chain), GSMUA_ Achr3G26220_001 (Probable xyloglucan endotransglucosylase/hydrolase protein 7), GSMUA_Achr1G17220_001 (Stigma/stylar cysteine-rich adhesin), GSMUA_Achr 11G11670_001 (Calvin cycle protein CP12-2), GSMUA_Achr6G01490_001 (Unknown), GSMUA_Achr9G12290_001 (Unknown) and GSMUA_Achr9G26820_001 (Unknown) with down regulation. These 17 genes and their regulations appear likely attributed to the cold tolerance for plantain. Besides aforementioned 66 differential genes, 172 more DEGs were found only in banana for 6 h of cold stress, which are expected to be involved in cold stress for banana only.

### Functional classification of cold stress-related DEGs

The functional classification of DEGs in banana and plantain was further examined to explore the pattern of transcriptome regulation that occurs during cold stress. Genes matching well-characterized proteins or proteins with putative functions were grouped using the gene ontology (GO) and summarized in Additional file 4: Table S4 and Table 3. The majority of DEGs identified in this study are in the categories regulation of transcription, response to stress, transport, protein modification, nucleosome assembly, cell wall organization and biogenesis, signal transduction, oxidation reduction, RNA modification, cell redox homeostasis, etc. No obvious difference of GO classification was observed for those DEGs from either banana (Additional file 4: Table S4) or plantain (Table 3), despite the fact that few DEGs in each GO category were identified in plantain than in banana.

### Validation of the DEGs by quantitative RT-PCR analysis

**Figure 3:**
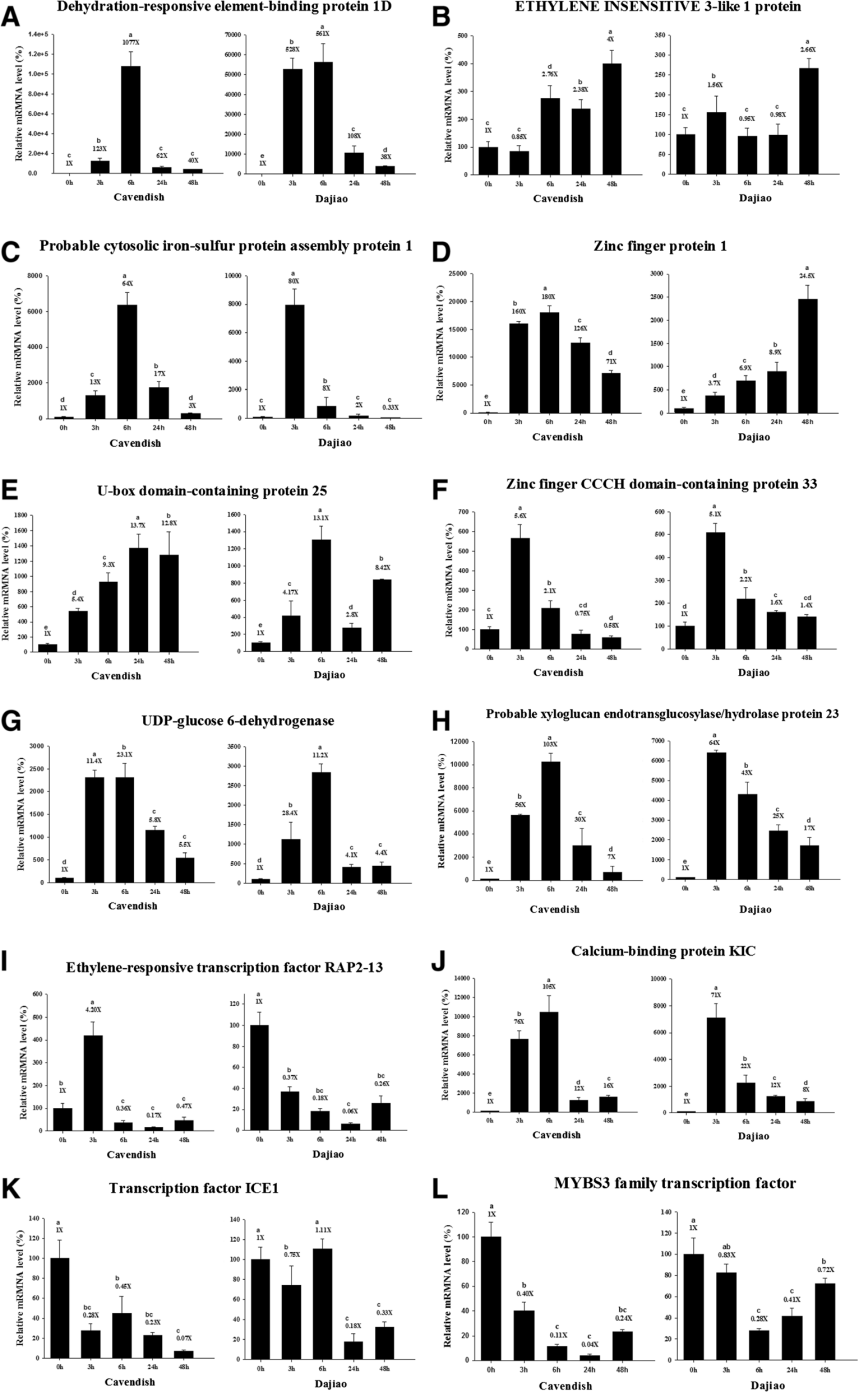
Relative mRNA levels of 12 DEGs in banana and plantain seedlings were determined by quantitative RT-PCR analyses. Six-leaf stage seedlings were incubated at 10°C for the indicated time. Transcript abundances of genes encoding dehydration-responsive element-binding protein 1D **(A)**, ethylene insensitive 3-like 1 protein **(B)**, probable cytosolic iron-sulfur protein assembly protein 1 **(C)**, zinc finger protein 1 **(D)**, U-box domain- containing protein 25 **(E)**, zinc finger CCCH domain-containing protein 33 **(F)**, UDP-glucose 6-dehydrogenase **(G)**, probable xyloglucan endotransglucosylase/hydrolase protein 23 **(H)**, ethylene-responsive transcription factor RAP2-13 **(I)**, calcium-binding protein KIC **(J)**, transcription factor ICE1 **(K)** and MYBS3 **(L)** from both banana and plantain were determined and compared across the time course of cold stress. Data represent means ± SD in four replicates (n = 4). The different lowercase letters labeled above columns indicate a significant difference at p ≤ 0.05 between the columns by Duncan’s test using SPSS statistical software (version 16.0, SPSS Inc. Chicago, IL). The columns with the same letters mean no significant difference (p > 0.05) between each other.

Plantain leaves showed significant phenotypic difference from banana after 3 h cold treatment at 10°C, we suspect that early response genes of plantain are closely associated with its cold resistance, thus 10 DEGs of plantain identified in this study after 3 h cold treatment and 2 critical cold-response homologous genes in arabidopsis ICE1 and rice MYBS3 were selected for quantitative RT-PCR analysis. Two additional extended time points for 24 and 48 h of cold stress were also measured in parallel with initial 0, 3 and 6 h cold stressed samples in both banana and plantain. The primers of selected genes are listed in Additional file 5: Table S5. 25S ribosomal RNA gene was used as reference gene for data normalization according to Van den Berg et al. [25]. The quantitative RT-PCR results from banana and plantain are shown in Figure 3. The expression profiles of all 12 detected genes show the same trend and consistent results between the RT-PCR and the Solexa-sequencing methods. For 24 and 48 h of cold stress, 6 out of 10 DEGs displayed the same down regulation in both banana and plantain, including GSMUA_Achr7G05900_001 (Dehydration-responsive element-binding protein 1D), GSMUA_Achr9G07610_001 (Probable cytosolic iron-sulfur protein assembly protein 1), GSMUA_Achr6G32910_001 (Zinc finger CCCH domain- containing protein 33), GSMUA_Achr7G26580_001 (UDP-glucose 6-dehydrogenase), GSMUA_ Achr3G04360 _001 (Ethylene-responsive transcription factor RAP2-13), GSMUA_Achr3G23000_001 (Calcium-binding protein KIC). However, 4 of them showed opposite changes between banana and plantain, including GSMUA_Achr6G25670_001 (U-box domain-containing protein25), GSMUA_Achr8G21550_001 (Ethylene insensitive 3- like 1 protein), GSMUA_ Achr2G13410_001 (Zinc finger protein 1) and GSMUA_Achr 3G05220_001 (Probable xyloglucan endotransglucosylase/hydrolase protein 23). Strikingly, there is a remarkable difference of *ICE1* and *MYBS3* expression profile between plantain and banana in response to cold stress. The *ICE1* was significantly down-regulated (decreased by 4-fold) under cold stress at early stage (3 h) in banana, but its decrease (by ~5-fold) didn’t appear until at 24 h in plantain (Figure 3K). Although the *MYBS3* was down-regulated similarly at early stage by 9-fold and 4-fold in banana and plantain respectively, at 6 h of cold stress (Figure 3L), it can be recovered much quickly in plantain than banana. With the extended cold stress, the plantain *MYBS3* was rapidly recovered to the level of 41% (at 24 h) and 75% (at 48 h), while the banana *MYBS3* was further decreased to 4% at 24 h and then recovered to 24% at 48 h of cold stress. The considerably different expression profiles of the two important transcriptional factors with a remarkable time delayed response in banana versus plantain suggest that the specific time course-based expression of *ICE1* and *MYBS3* in plantain might be related to its cold tolerance.

## Discussion and conclusions

In our previous study on the temporal responses of plantain to cold stress using quantitative proteomics analysis, we have revealed that antioxidation mechanisms contribute to cold tolerance in plantain at the global proteome level [10]. However, due to the limitation of current technologies in overcoming the issue of the wide dynamic range in proteomics samples, quantitative proteomics analysis often misses the detection of many low abundance proteins, yielding only 10-30% proteome coverage for any given non-model species. Obviously this will reduce the number of low abundance proteins identified during global functional studies. Given the above consideration and to focus on the molecular mechanisms involving low abundance genes on the cold-tolerance of plantain, a comparative transcriptomics analysis of cold-sensitive banana and cold-tolerant plantain was conducted by RNA-Seq and real time RT-PCR with time course of cold treatment.

After 48 h of cold stress at 10°C for banana and plantain seedlings, both phenotype and electrolyte leakage analyses clearly indicate that plantain seedlings exhibited a more robust cold tolerance than banana seedlings (Figure 1). It is this phenotypic difference in the cold response between banana and plantain that provides an excellent model system allowing us to study differential gene expression in response to cold stress, as well as to elucidate the potential different cold responsive mechanisms between banana and plantain. RNA-Seq analysis shows that 10 and 68 DEGs are identified for 3 h and 6 h of cold stress respectively in plantain, while the equivalent DEGs are 40 and 238 being identified in banana (as shown in Figure 2), indicating banana is much more sensitive to cold stress than plantain. GO classification analysis shows the majority of DEGs identified in this study belong to the following 11 categories: regulation of transcription, response to stress, transport, protein modification, nucleosome assembly, cell wall organization and biogenesis, signal transduction, oxidation reduction, RNA modification, cell redox homeostasis, etc. Since there is no difference of GO classification found between banana (Additional file 4: Table S4) and plantain (Table 3), it suggests that cold stress appears to have a broad range of impacts on cellular activities. However, when we further performed the pathway analysis for some specific DEGs either found uniquely in plantain particularly or detected at variable cold stress time points in both banana and plantain, we found that many DEGs and their associated pathways are likely to be involved in cold-tolerance of plantain. Below we discuss intensively some of the important pathways and their possible mechanisms associated with the observed cold tolerance in plantain.

### *ICE1* and *MYBS3* pathways

*ICE* gene was initially identified and isolated from *Arabidopsis thaliana*. The function of *AtICE1* is to activate gene expression of C-repeat binding factor (*CBF3*) to enhance the cold resistance of *Arabidopsis* [26]. In *Oryza sativa*, three novel MYB proteins (MYBS1, MYBS2 and MYBS3*)* containing one highly conserved DNA-binding domain mediate sugar and hormone regulation by promoting gene expression of alpha-amylase [27]. Recent studies reveal that *MYBS3* is also involved in adaptation of cold stress in rice. Compared to the *ICE1*, *MYBS3* functions particularly in late stage of plant for adaptation of cold stress. For example, transgenic rice overexpressing *MYBS3* can resist cold stress at 4°C for a week [14]. In the early stage of cold response, the transient activation of *αAmy3* expression by *CBF* allows hydrolysis of reserved starch to meet the immediate need for a carbon source and energy to combat the cold shock, while the subsequent suppression of *αAmy3* expression by *MYBS3* allows rice to conserve carbohydrates until regrowth is allowed at elevated temperatures [14]*.* In this study using RNA-Seq analysis for 0, 3, and 6 h of cold stressed samples, although both *ICE1* and *MYBS3* genes were detected, neither was identified as DEG. This is not surprising, because *ICE1* requires activation, which is regulated by post-translational modifications via phosphorylation and sumoylation of the *SIZ1* gene [28,29]. Thus, *ICE1* is likely not regulated at the transcriptional level. Since *MYBS3* plays a role in cold adaptation at late stage of plant growth, *MYBS3* is expected not present in early time points from cold stressed samples. In fact, we did identify both genes by RNA-Seq analysis at a low level with relatively large variation across the 4 replicate samples. Given the well-known functions of both *ICE1* and *MYBS3* genes in plant resistance to cold stress, we decided to use quantitative RT-PCR for assessing their changes with the extended time points at 24 and 48 h of cold stress in this study. The results indicate that although there is no significant change of *ICE1* in plantain at 3 and 6 h cold stress, both an upstream gene: GSMUA_Achr9P04380_001(*MpSIZ1*, 68% homology with arabidopsis *SIZ1*) and a downstream gene: GSMUA_ Achr7 G05900_001 (*CBF*) in an *ICE1-CBF-COR* pathway, were up-regulated (Additional file 6: Figure S1; Figure 3A). Since the *ICE1-CBF-COR* is a well-known pathway involved in plant cold-tolerance [30], the up-regulation of *CBF* is therefore, expected to facilitate the cold-tolerant pathway. A 3.5-fold increase of COR47 protein found in our proteomics analysis of plantain for 6 h under cold stress at 8°C [10] supports this observation with the *ICE1-CBF-COR* pathway being activated in response to the cold stress in this study. Interestingly, at 24 h of cold stress, both plantain *ICE1* and *CBF* were down regulated. Meanwhile, the *MYBS3* gene had almost completely recovered (from early down-regulation in response to the cold stress), which may offset the down-regulated *ICE1-CBF-COR* pathway as a means of sustaining the cold resistance. In banana, however both *ICE1* and *MYBS3* were significantly decreased under cold stress. The *CBF* response to cold stress in banana is significantly slower than that in plantain, until 6 h of cold stress, when the banana *CBF* gene was up-regulated, only to go down again at 24 h of cold stress. Consistently, the time delayed response of *MYBS3* gene to the cold stress was also observed in banana. However, at 48 h of cold stress, the banana *MYBS3* gene appeared recover from its initial cold suppression. Based on the observation of slow response to the cold stress in banana for both *ICE1* and *MYBS3* as the two critical transcriptional factors responsible for early and late stage respectively against cold stress, we suspect that the relatively low expression of these two key genes along with their delayed response to the cold stress are among the main reasons for cold sensitive banana.

### Signals transduction

As an important second messenger, Ca^2+^ plays a vital role in the plant cold-stress response. The concentration of Ca^2+^ inside the cell increases rapidly during cold stress, followed by a number of signals mediated by a series of protein phosphorylation cascades [31]. During rice domestication, an amino acid mutant of COLD1 from Met^187^/Thr^187^ to Lys^187^ in *japonica* cultivars was found to enhance cold tolerance partly because it facilitates the formation of an appropriate Ca^2+^ signal by increased Ca^2+^ concentration [15]. Interestingly, our study shows that most of the DEGs involving in signal transduction are indeed related to the calcium-dependent signal pathway, such as orthologs of *CIPK* (*CBL*-interacting protein kinase), *KIC* (Kinesin-like calmodulin binding protein) and *CML* (Calmodulin-like protein). All three genes in banana and plantain were up-regulated in response to cold stress. In addition, overexpression of *OsMSR2,* a novel rice calmodulin-like gene, improves resistance of drought and salt and increases *ABA* sensitivity in *Arabidopsis* [32]. In this work, we found the dramatically increased expression of *CML7* (*GSMUA_Achr6G35120_001*), an ortholog of *OsMSR2,* in response to cold stress in plantain but not in banana, suggesting that there is a more efficient response of the Ca^2+^ signal transduction pathway in plantain so that it can quickly and effectively regulate downstream signaling and gene expression in response to cold stress.

As a signal molecule, auxin plays an important function under various abiotic stress conditions [33]. Intriguingly, compared to the control group (0 h), the expression of GSMUA_Achr4G16550_001 (Auxin-induced in root cultures protein 12, *AIR12*) was increased by 9 fold in 6 h cold treatment in plantain, while no differential expression of the gene was detected in banana. On the plasma membrane, AIR12 is able to receive auxin signal to promote lateral root morphogenesis and participates in the decomposition of glucose [34], while it has been proven that the content of soluble sugar and acid can affect the plant’s cold tolerance [35,36]. Our results indicate that the increased expression of *AIR12* in plantain could facilitate the growth of plantain root and the content of sugar and acid, which might be attributed to the better resistance to cold.

### Transcription regulation

In this study, we have identified 10 differentially expressed transcription factors in response to cold stress in banana and plantain, including *NAC*, *ERF* (Ethylene response transcription factors), *DREB* (Dehydration-responsive element-1binding protein), *MYB,WRKY, C3H33*, *EIL1* (Ethylene insensitive 3-like 1 gene)*, ADO3* (Adagio-like gene 3), *ZFP* (Zinc finger protein) and *RPOA* (DNA-directed RNA polymerase subunit alpha). NAC transcription factors were up-regulated in banana and plantain after 6 h cold stress, and over-expression of *SNAC2* was reported to increase cold and salt resistance in rice [37], suggesting that NACs are likely to participate in cold response in banana and plantain. Effects of ethylene on plants under cold stress have recently been recognized, the *ERF* transcription factors which related to ethylene, were up-regulated in banana and plantain after 6 h cold stress. Overexpression of transcription factor *TERF2*/*LeERF2* in tobacco and tomato was reported to result in cold tolerance by facilitating ethylene biosynthesis [38], which supports the important role of *ERF* in plant cold tolerance. *DREB* transcription factors, with a conservative *AP2/EREBP* domain, are involved in regulation of stress-related gene in response to the external environment, through binding *DRE CIS*-acting elements [39]. Our data shows that overexpression of *DREB1* occurs at 3 h of cold stress in plantain versus 6 h in banana, suggesting that the early response of *DREB1* may be critical in cold-tolerant plantain. Due to a large number of *MYB* transcription factor found in plants such as *Arabidopsis thaliana* and *Zea mays*, an increased amount of research has been focused on its role in transcriptional regulation and its impact on a broad range of physiological functions, e.g., overexpression of rice *MYB* genes (*OsMYB3R-2* and *OsMYB4*) enabled to significantly enhanced the cold tolerance of transgenic *Arabidopsis thaliana* [40]. In our work,we found GSMUA_Achr8G25220_001-a MYB transcription factor increased after 6 h cold stress while it was undetectable at 0 h in both banana and plantain, indicating that this gene is a cold-induced gene. The change of this *MYB* gene could affect downstream related genes. In *Arabidopsis thaliana*, the *WRKY* gene family contains 17 genes induced by cold stress, most of which are activated in its early stages [41,42]. After 6 h cold stress, the *WRKY* genes showed up-regulation, among them, GSMUA_Achr7G05200_001 was overexpressed whereas it was undetectable at 0 h, indicating it is also a cold-induced gene. In plantain, only one cold-induced *WRKY* gene-GSMUA_Achr6G15840_001 was detected after 6 h of cold stress. However, its expression was much higher than that in banana, suggesting that this *WRKY* gene may contribute to the cold-resistance of plantain. It has been reported that zinc-finger proteins are involved in resistance to adversity in plant [43]. For example, *GmZF1* from soybean was found to enhance the tolerance of *Arabidopsis* to cold stress by expression of cold-regulation genes in the transgenic *Arabidopsis* [44]. Strikingly, banana *ZFP1* is not up-regulated until 24 h of cold stress and starts to decrease at 48 h, while plantain *ZFP1* is constantly up-regulated beyond 48 h of cold stress, suggesting *ZFP1* may play a role in late stages of cold stress (Figure 3D). Expression of *EIL1* in plantain reached ~11-fold higher induction at 3 h cold treatment and then decreased back to initial levels at 48 h. In banana, the *EIL1* was up-regulated by ~9-fold at 6 h and further increased to ~13-fold at 48 h of cold treatment (Figure 3B). As a primary transcription factor in ethylene signal transduction, *EIL* regulates transcription of downstream genes to complete the ethylene response [45]. Given the higher expression of *EIL* at very early stages and its rapid decrease in response to cold stress in plantain, we suspect that rapid and early activation of the ethylene signal transduction pathway appears to play a critical role in the rapid response to cold stress allowing early protection in plantain.

### Genes associated with stress response

Plants often activate similar pathways in response to different types of abiotic stress. In this cold stress study, we found quite a few genes related to stress response in both banana and plantain, such as *STHY* (Probable salt tolerance-like protein), *P2C32* (Probable protein phosphatase 2C 32), ubiquitin-protein ligase genes, *THIL-2* (Thiamine thiazole synthase 2), *GRP*(Glycine-rich RNA-binding protein) and *DRT100* (DNA-damage-repair/toleration protein), *etc.* Those genes showed similar patterns of expression in response to cold treatment in banana and plantain, suggesting the stress response genes share some common response pathways in both species. However, it should be noted that some of the DEGs are located in the chloroplast of plantain only, such as *ELIP*, *CTL1*, *SCA* and *MKS1*. In *Rhododendron catawbiens*, expression of *RcELIP* (early light-induced protein) resulted in adaptive responses to cold and high light in winter-adapted rhododendron leaves, suggesting a critical role of *ELIP* in protection of photosynthetic apparatus from these stresses [46]. In *Arabidopsis thaliana*, *CTL1* (Chitinase-like protein) is involved in altering the architecture of the root system in response to multiple environmental conditions [47]. As an *MAP* kinase substrate, *Arabidopsis MKS1* may be involved in *MPK4*-regulated activation of plant defense by coupling the kinase to specific *WRKY* transcription factors [48]. Due to the expression of *ELIP*, *CTL1*, *SCA* and *MKS1* induced by cold treatment exclusively in plantain (not in banana), we believe that plantain possesses the highly superior aspects of photosynthesis, root architecture and plant defense regulated by MPK, which makes plantain broadly resist to abiotic stress resulting in a better cold tolerance.

### Other DEGs involved in functional regulations

In addition to the above four categories, we found some DEGs in banana and plantain are also involved in protein/RNA modification, cell wall composition and occurrence, transit, nucleosome assembly, cellular redox balance, iron-sulfur cluster assembly, protein hydrolysis, oxidation and reduction, copper ion balance, photosynthesis, respiration and sugar stimulus response. But again some of the cold responsive DEGs are found exclusively in plantain, including *MTP3* (a 3-fold increase at 6 hr cold stress) involved in copper ion balance, *RBCS* (a 10-fold decrease at 6 hr) functioning in photosynthesis and respiration in chloroplast, *CP122* (an 80-fold decrease at 6 hr) involved in sugar stimulus response in chloroplast, and *UBQ11* (a 5-fold increase at 6 hr) functioning in protein modification. In yeast, *PutMT2* (type 2 metallothionein-like protein in *Puccinellia tenuiflora*) has been verified to play a critical role in improving tolerance to metal and reactive oxygen species [49]. Thus the up-regulation of *MTP3* would allow plantain for improved tolerance to the increased reactive oxygen species (ROS) due to the cold stress [10]. In plant, *RBSC* encodes the small subunit of Rubisco (Ribulose-1, 5-bisphosphate carboxylase/oxygenase) involved in the first major step of carbon fixation and the *RBSC* gene family functions to yield sufficient Rubisco content for leaf photosynthetic capacity [50]. As the expression of *RBCS* was highly repressed in plantain, we speculate that the protection mechanism is activated in response to cold treatment in chloroplast in order to decrease the production of ROS through the weakened photosynthesis. *Arabidopsis CP12* forms a complex with *GAPDH* (Glyceraldehyde-3- phosphate dehydrogenase) and *PRK*(Phosphoribulokinase) in regulating TCA cycle [51]. A dramatic repression of *CP122* suggests that plantain must reduce the photosynthesis in response to cold stress. This is consistent with the decreased *RBCS* but as another pathway for the self-protection mechanism. *UBQ* (poly-ubiquitin gene) is involved in protein hydrolysis in the ubiquitin-proteasome pathway. In order to compensate cells for ubiquitin molecules in signal transmission, expression of poly-ubiquitin gene is increased in response to stress [52]. Given the increased expression of *UBQ* in response to cold treatment, we suspect that UBQ might be involved in stress response in plantain.

**Figure 4:**
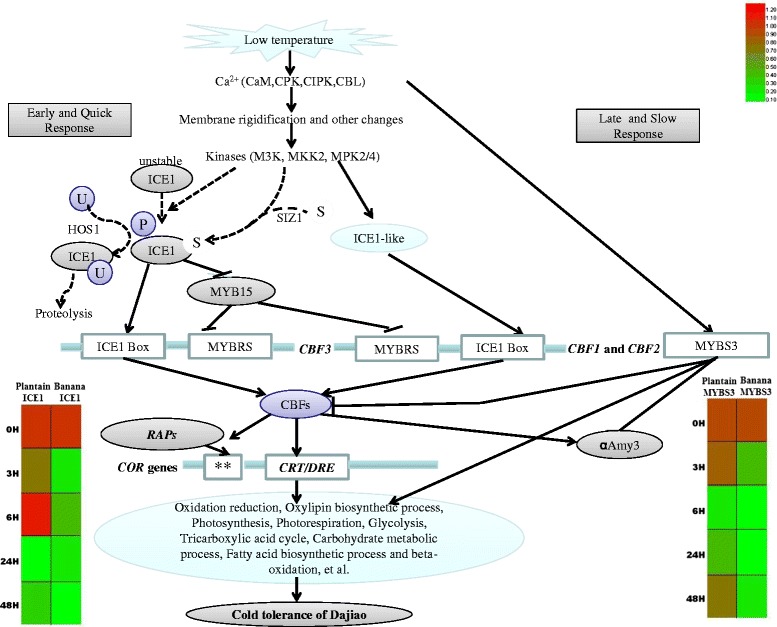
A schematic diagram of cold-tolerance transcriptional network in plantain, adapted initially from V. Chinnusamy et al. (2007) [4] and C.F. Su et al. (2010) [14] and revised based on this study. At the early stage of cold stress, plantain cells probably sense low temperatures through membrane rigidification and/or other cellular changes, which might induce a calcium signature and activate protein kinases necessary for cold tolerance. Constitutively expressed ICE1 is activated by cold stress through sumoylation and phosphorylation. Sumoylation of ICE1 is critical for ICE1-activation of transcription of *CBFs* and repression of *MYB15*. CBFs regulate the expression of *COR* genes that confer cold tolerance. The expression of *CBFs* is negatively regulated by MYB15. HOS1 mediates the ubiquitination and proteosomal degradation of ICE1 and, thus, negatively regulates CBF regulons. CBFs can constitutively regulate the expression of downstream cold-responsive transcription factor genes *RAPs,* which might control sub-regulons of the *CBF* regulon. CBFs also activate *αAmy3* expression to hydrolyse reserved starch. At the late stage of cold stress,MYBS3 inhibits *CBFs* and *αAmy3* expression. The effective coordination across the early and late stages of cold stress by at least two different regulatory pathways appears to efficiently regulate the following metabolic pathways including oxidation reduction, oxylipin biosynthetic process, photosynthesis, photorespiration, glycolysis, tricarboxylic acid cycle, carbohydrate metabolic process, fatty acid biosynthetic process and beta-oxidation. The rapid activation and selective induction of ICE1 and MYBS3 cold tolerance pathways in plantain, along with expression of other cold-specific genes, may be one of the main reasons that plantain has higher cold resistance than banana (Heatmaps show the expression of *ICE1* and *MYBS3* in banana and plantain under cold stress). Broken arrows indicate post-translational regulation; solid arrows indicate activation, whereas lines ending with a bar show negative regulation; the two stars (**) indicate unknown cis-elements. P, phosphorylation; S, SUMO; U, ubiquitin.

In summary,a global, comparative transcriptomic profile of banana and plantain in response to cold treatment has been intensively investigated by transcriptome sequencing (0 h, 3 h and 6 h) and quantitative RT-PCR (0 h, 3 h, 6 h, 24 h and 48 h). At the transcriptomic level, both plantain and banana share similar profiles for the majority of DEGs in time course response to cold treatment. Many of the DEGs have been reported and involved in the development of cold resistance or other stress resistance in plant, such as: *CIPK, KIC, CML* (involved in signal transduction), *NAC, ERF, DREB, MYB, WRKY, C3H33, ADO3, RPOA* (involved in transcriptional regulation), *STHY*, *P2C32*, ubiquitin-protein ligase genes, *THIL-2*, *GR* and *DRT100* (involved in stress response), *etc*. Despite the similar profiles of DEGs found in banana and plantain, several dozen DEGs involved in several different pathways were successfully identified and validated only in cold-tolerant plantain. For example, the cold-tolerant ICE1 and MYBS3 pathways were rapidly activated and switched in early (3 h) and late (24 h) stages respectively, of response to cold treatment in plantain. While in cold-sensitive banana, no such equivalent regulation for ICE1-CBF-COR pathway was found and slow response to MYBS3 pathway responsible for the cold stress was observed. Thus, the low expression of the two key genes along with their delayed response to the cold stress may be one of the main reasons for cold sensitivity of banana. The cold-tolerant plantain also appears closely related to expressions of several specific genes such as *CML7*, *AIR12* (for signal transduction), *ZFP1*, *EIL1* (for transcriptional regulation), *ELIP, CTL1, SCA, MKS1* (for stress response), *MTP3* (for copper ion balance), *RBCS* (for photosynthesis and photorespiration), *CP122* (sugar stimuli response) and *UBQ11* (protein modification). Combined with previous research, we rationalize that in early stage of cold stress response, changes in cell membrane phase trigger modification of actin cytoskeleton, which activates ICE1-CBF-COR metabolic pathways by Ca^2+^ and phosphorylation signaling pathways. Also it triggers an early response via the ethylene signal transduction pathway. In late stage of cold stress, expression of *MYBS3* begins to recover. *MYBS3* is involved in coordination with the expression of multiple genes for regulation of oxidation/reduction, oxylipin biosynthetic process, photosynthesis, photorespiration, glycolysis, tricarboxylic acid cycle, carbohydrate metabolic process, fatty acid biosynthetic process and beta-oxidation, enhanced adoption to cold tress (Figure 4). The next questions need to be answered are what causes the observed different expression profiles between ICE1 and MYBS3 pathways in plantain? How do both coordinate together for effectively regulating cold-resistance? Addressing these questions will further enhance the understanding on signaling and metabolic pathways of cold tolerance in plantain, but also conducive to nurturing new varieties of cold tolerant banana cultivars.

## Methods

### Plant materials

Seedlings of the cold-tolerant plantain (*Musa spp*. Dajiao; ABB Group) and the cold-sensitive banana (*Musa spp*. Cavendish; AAA Group) with a uniform growth stage were obtained from Institute of Fruit Tree Research, Guangdong Academy of Agricultural Sciences, Guangzhou, P. R. of China.

### Experimental design and cold stress treatment

The main objective in this study was to compare cold-response genes between cold-sensitive banana and cold-tolerant plantain in order to gain a better understanding of the underlying signal transduction and the molecular mechanisms of cold tolerance plantain at transcriptomics level. Mature plantain plants can tolerate temperatures of 0–4°C. In our previous quantitative proteomics analysis, we found 219 differentially expressed proteins in plantain seedlings with cold stress at 8°C for only 6 hours and the plant leaves drooped with wilting symptoms after 24 h of cold treatment [10]. To identify some sensitive and early cold-response genes in both banana and plantain, in this study we increased the cold-treatment temperature to 10°C and reduced exposure time at 0, 3 and 6 hours respectively. The comparative transcriptomic analysis was conducted for the cold stressed seedlings, followed by large scale identification and functional categorization of the differentially expressed, early responsive genes. Furthermore, quantitative real time-PCR was carried out to validate the early cold-responsive transcriptomic results and some late-responsive genes with the extended time of cold treatment for 24 and 48 hours.

Seedlings were grown in a growth chamber at 30/28°C (day/night), a photon flux density of 240 μmol m^−2^ s^−1^ throughout a 12-h photoperiod, and a relative humidity of 60-80%. Six-leaf stage seedlings were used in the experiment. Low temperature treatments were started at 12:00 AM on the first day by setting the temperature to 10°C, which was reached about 30 min later. The first young leaf was detached from the top of each of the 5 plants at each time point (10°C for 0, 3 and 6 h) for each biological replicate. The leaves from the 5 plants were cut into pieces (1.5 × 1.5 cm) and mixed well. Aliquots of the mixed tissues were frozen in liquid N_2_ and stored at −80°C until use.

### Measurements of relative electrolyte leakage

The leaves were cut into 1-cm segments and washed three times with ultrapure water. The segments were placed in tubes containing 5 mL of ultrapure water and incubated at 25°C. Two hours later, the electrical conductivity of the bathing solution (*L1*) was measured. Then the tubes were incubated at 100°C for 20 min and subsequently at 25°C for 1 h, and the electrical conductivity (*L2*) was measured again. The relative electrolyte leakage was calculated by the formula (*L1-L0*)/(*L2-L0*) × 100 (L0, conductivity of ultrapure water) [53]. Five replicates were performed for each sample.

### Sample preparation, cDNA library construction and illumina sequencing

Total RNA was extracted from banana and plantain leaves (Four biological replicates of Cavendish and Dajiao seedlings at 10°C for 0, 3 and 6 h) using plant total RNA isolation kit (Tiandz Inc; Beijing, China). RNA degradation and contamination was monitored on 1% agarose gels; RNA purity was checked using the NanoPhotometer® spectrophotometer (IMPLEN, CA, USA) and RNA integrity was assessed using the Bioanalyzer 2100 system (Agilent Technologies, CA, USA). A total of 3 μg RNA per sample was used as input material in RNA sample preparations for subsequent cDNA library construction. All 24 samples had RIN values above 8.0. Sequencing libraries were generated using Illumina TruSeq™ RNA Sample Preparation Kit (Illumina, San Diego, USA) following manufacturer’s recommendations and four index codes were added to attribute sequences to each sample. Briefly, mRNA was purified from total RNA using poly-T oligo-attached magnetic beads. Fragmentation was carried out using divalent cations under elevated temperature in Illumina proprietary fragmentation buffer. First strand cDNA was synthesized using random oligonucleotides and SuperScript II. Second strand cDNA synthesis was subsequently performed using DNA Polymerase I and RNase H. Remaining overhangs were converted into blunt ends via exonuclease/polymerase activities and enzymes were removed. After adenylation of 3′ ends of DNA fragments, Illumina PE adapter oligonucleotides were ligated to prepare for hybridization. In order to select cDNA fragments of preferentially 200 bp in length the library fragments were purified with AMPure XP system (Beckman Coulter, Beverly, USA). DNA fragments with ligated adaptor molecules on both ends were selectively enriched using Illumina PCR Primer Cocktail in a 10 cycle PCR reaction. Products were purified (AMPure XP system) and quantified using the Agilent high sensitivity DNA assay on the Agilent Bioanalyzer 2100 system. The clustering of the index-coded samples was performed on a cBot Cluster Generation System using TruSeq PE Cluster Kit v3-cBot-HS (Illumina) according to the manufacturer’s instructions. After cluster generation, the library preparations were sequenced on an Illumina Hiseq 2000 platform and 100 bp fragment reads were generated. The data was submitted into the NCBI SRA database (Accession No. SRP047347).

### Sequence annotation

Raw data (raw reads) of fastq format were firstly processed through in-house perl scripts. In this step, clean data (clean reads) were obtained by removing reads containing adapter, reads containing ploy-N, and low quality reads from raw data. At the same time, Q20, Q30, GC content and sequence duplication level of the clean data were calculated. All the downstream analyses were based on the clean data with high quality. Reference genome and gene model annotation files were downloaded from the banana genome website (http://banana-genome.cirad.fr/content/download-dh-pahang) directly. Index of the reference genome was built using Bowtie v0.12.8 and paired-end clean reads were aligned to the reference genome using TopHat v1.4.0. HTSeq v0.5.3 was used to count the reads numbers mapped to each gene. And then RPKM of each gene was calculated based on the length of the gene and reads count mapped to this gene.

### Identification of Differentially Expressed Genes (DEGs)

Differential expression analysis was performed using the DEGSeq R package (1.12.0). *P*-values were adjusted using the Benjamini & Hochberg method. Corrected *P*-value of 0.05 and log2 (fold change) of 1 were set as the threshold for significantly differential expression. Gene Ontology (GO) enrichment analysis of differentially expressed genes was implemented by the GOseq R package, in which gene length bias was corrected. GO terms with corrected *P*-value less than 0.05 were considered significantly enriched by differential expressed genes.

### Quantitative RT-PCR

Total RNA was isolated from banana and plantain leaves in four biological replicates at five different time points as described above. The resulting RNA (1 μg) was used as a template for first-strand cDNA synthesis using ReverTra Ace (Toyobo, Osaka, Japan) with random hexamers according to the manufacturer’s instructions. Primer pairs for real-time quantitative PCR (see Additional file 5: Table S5 online) were designed using Primer Premier 5.0 (Premier Biosoft, Palo Alto, USA). The PCR reaction consisted of 10 μL of 2 × SYBR Green PCR Master Mix (Toyobo), 200 nM primers, and 2 μL of 1:40-diluted template cDNA in a total volume of 20 μL. No template controls were also set for each primer pair. Real-time PCR was performed employing the DNA Engine Option 2 Real-Time PCR Detection system and Opticon Monitor software (Bio-Rad, USA).

### Availability of supporting data

The sequencing raw data of this article have been deposited in a SRA database at the NCBI -http://www.ncbi.nlm.nih.gov/Traces/sra_sub/sub.cgi?subid=321444&from=list&action=show:submission.

## Additional files

Additional file 1: Table S1. The raw data quality of libraries. Additional file 2: Table S2. Original data of Banana gene expression profiles under cold stress. Additional file 3: Table S3. Original data of Plantain gene expression profiles under cold stress. Additional file 4: Table S4. Primary functional classification on differential genes of Banana with fold changes > or < 2-fold. Additional file 5: Table S5. List of primers used for the Real-time PCR. Additional file 6: Figure S1. Relative mRNA levels of E2 SUMO-protein ligase SIZ1 in banana and plantain seedlings were determined by quantitative RT-PCR analyses. Six-leaf stage seedlings were incubated at 10°C for the indicated time. Transcript abundances of genes encoding E2 SUMO-protein ligase SIZ1 from both banana and plantain were determined and compared across the time course of cold stress. Data represent means ± SD (n = 4). The different lowercase letters labeled above columns indicate a significant difference at p ≤ 0.05 between the columns by Duncan’s test using SPSS statistical software (version 16.0, SPSS Inc. Chicago, IL). The columns with the same letters mean no significant difference (p > 0.05) between each other.

## Abbreviations

RPKM: Reads per kilobase of exon model per million mapped reads
DEG: Differentially expressed gene
GO: Gene ontology
*ICE1*: Inducer of *CBF* expression
*CBF/DREB1*: C-repeat/dehydration-responsive element binding factor
*MYBS3*: DNA-binding repeat MYB transcription factor S3
*RCI*: Rare cold-induced gene
*ELIP*: Early light-induced protein
*CTL1*: Chitinase-like protein 1
*SCA*: Stigma/stylar cysteine-rich adhesion
*MKS1*: MAP kinase substrate 1
*MTP3*: Metallothionein-like protein type 3
*RBCS*: Ribulose bisphosphate carboxylase small chain
*CP122*: Calvin cycle protein CP12-2
*AIR12*: Auxin-induced in root cultures protein 12
*UBQ11*: Polyubiquitin 11
*GAPDH*: Glyceraldehyde-3-phosphate dehydrogenase
*PRK*: phosphoribulokinase
SUMO: Small ubiquitin related modifier
SOD: Superoxide dismutase
MDA: Malondialdehyde
ROS: Reactive oxygen species
RIN: RNA Integrity Number
*ERF*: Ethylene-responsive transcription factor
*CBL*: Calcineurin B-like protein
*CIPK*: CBL-interacting serine/threonine-protein kinase
*CML*: Calmodulin-like protein
*COR*: Cold regulated gene
*EIL*: Ethylene insensitive 3-like 1 protein
*ADO*: Adagio-like protein
*STHY*: Probable salt tolerance-like protein
*ZFP*: Zinc finger protein
*Thi1-2*: Thiamine thiazole synthase 2
*GRP*: Glycine-rich RNA-binding protein
*DRT100*: DNA-damage-repair/toleration protein DRT100
*MPK4*: Mitogen-activated protein kinases 4
*MAP3K*: MAP kinase kinase kinase
*HOS1*: RING-finger E3 ubiquitin ligase
*α-Amy3*: α-amylase3
