# Supplementary material for: Comparative transcriptomics analysis reveals difference of key gene expression between banana and plantain in response to cold stress
Source: BMC Genomics. 2015 Jun 10;16(1):446. doi: 10.1186/s12864-015-1551-z (PMC4461995; doi:10.1186/s12864-015-1551-z)
Supplement: Additional file 4: Table S4. — Primary functional classification on differential genes of Banana with fold changes > or < 2-fold. [file 12864_2015_1551_MOESM4_ESM.doc]

Additional file 4: **Primary functional classification on DEGs of banana with more than 2-fold changes**

| **Gene_id** | **RPKM Cavendish_0h** | **RPKM Cavendish_3 h *^a^*** | **RPKM Cavendish_6 h** | **Log2Fold Change 3 h** | **Log2Fold Change 6 h** | **Gene name** |
| --- | --- | --- | --- | --- | --- | --- |
| Regulation of transcription |  |  |  |  |  |  |
| GSMUA_Achr5G07590_001 | 0.00 | 28.08 | 208.22 | Inf | Inf | sp|Q52QH4|NAC68_ORYSJ NAC domain-containing protein 68 |
| GSMUA_Achr4G02390_001 | 1.08 |  | 83.24 |  | 6.26 | sp|Q52QH4|NAC68_ORYSJ NAC domain-containing protein 68 |
| GSMUA_Achr7G21780_001 | 12.88 | 87.23 | 198.56 | 2.63 | 3.93 | sp|Q52QH4|NAC68_ORYSJ NAC domain-containing protein 68 |
| GSMUA_Achr6G32330_001 | 5.61 |  | 72.06 |  | 3.67 | sp|Q52QH4|NAC68_ORYSJ NAC domain-containing protein 68 |
| GSMUA_Achr5G07600_001 | 10.58 |  | 116.35 |  | 3.45 | sp|Q9FLJ2|NC100_ARATH NAC domain-containing protein 100 |
| GSMUA_Achr8G33590_001 | 0.00 |  | 69.30 |  | Inf | sp|Q40477|ERF4_TOBAC Ethylene-responsive transcription factor 4 |
| GSMUA_Achr8G06870_001 | 3.19 |  | 361.82 |  | 6.81 | sp|Q9LW49|ERF4_NICSY Ethylene-responsive transcription factor 4 |
| GSMUA_Achr7G03640_001 | 5.76 | 58.91 | 150.27 | 3.21 | 4.69 | sp|Q9LW49|ERF4_NICSY Ethylene-responsive transcription factor 4 |
| GSMUA_Achr9G11480_001 | 20.90 |  | 135.18 |  | 2.68 | sp|Q40477|ERF4_TOBAC Ethylene-responsive transcription factor 4 |
| GSMUA_Achr4G33270_001 | 2.20 |  | 162.82 |  | 6.19 | sp|Q9FE67|ERF80_ARATH Ethylene-responsive transcription factor 9 |
| GSMUA_Achr10G09150_001 | 0.99 |  | 37.21 |  | 5.22 | sp|Q9FE67|ERF80_ARATH Ethylene-responsive transcription factor 9 |
| GSMUA_Achr5G21050_001 | 2.39 | 51.76 | 100.67 | 4.32 | 5.38 | sp|Q40476|ERF1_TOBAC Ethylene-responsive transcription factor 1 |
| GSMUA_Achr2G07300_001 | 171.65 |  | 746.96 |  | 2.11 | sp|Q6K7E6|ERF1_ORYSJ Ethylene-responsive transcription factor 1 |
| GSMUA_Achr3G23710_001 | 4.81 |  | 199.66 |  | 5.36 | sp|Q9C5I3|ERF76_ARATH Ethylene-responsive transcription factor 11 |
| GSMUA_Achr7G06910_001 | 71.35 | 1280.14 | 2156.87 | 4.02 | 4.90 | sp|Q8VY90|EF105_ARATH Ethylene-responsive transcription factor ERF105 |
| GSMUA_Achr7G05410_001 | 6.29 |  | 143.98 |  | 4.50 | sp|Q8W3M3|RAP29_ARATH Ethylene-responsive transcription factor RAP2-9 |
| GSMUA_Achr3G01650_001 | 18.39 | 146.61 | 331.39 | 2.84 | 4.16 | sp|Q8H1E4|RAP24_ARATH Ethylene-responsive transcription factor RAP2-4 |
| GSMUA_Achr10G30440_001 | 6.59 |  | 98.67 |  | 3.89 | sp|Q8H1E4|RAP24_ARATH Ethylene-responsive transcription factor RAP2-4 |
| GSMUA_Achr8G25220_001 | 0.00 |  | 63.76 |  | Inf | sp|Q9FDW1|MYB44_ARATH Transcription factor MYB44 |
| GSMUA_Achr1G09750_001 | 14.98 |  | 236.34 |  | 3.97 | sp|Q9FDW1|MYB44_ARATH Transcription factor MYB44 |
| GSMUA_Achr4G20150_001 | 50.38 | 255.70 | 308.31 | 2.22 | 2.60 | sp|Q9FDW1|MYB44_ARATH Transcription factor MYB44 |
| GSMUA_Achr7G05200_001 | 0.00 |  | 23.17 |  | Inf | sp|Q9SK33|WRK60_ARATH Probable WRKY transcription factor 60 |
| GSMUA_Achr6G15840_001 | 3.25 |  | 165.71 |  | 5.66 | sp|Q9SKD9|WRK46_ARATH Probable WRKY transcription factor 46 |
| GSMUA_Achr6G05710_001 | 4.83 |  | 152.08 |  | 4.96 | sp|Q8H0Y8|WRK41_ARATH Probable WRKY transcription factor 41 |
| GSMUA_Achr11G10700_001 | 0.65 |  | 13.15 |  | 4.31 | sp|Q8S8P5|WRK33_ARATH Probable WRKY transcription factor 33 |
| GSMUA_Achr7G14140_001 | 4.15 |  | 78.60 |  | 4.22 | sp|Q8H0Y8|WRK41_ARATH Probable WRKY transcription factor 41 |
| GSMUA_Achr1G27980_001 | 31.50 |  | 158.26 |  | 2.31 | sp|Q9SUP6|WRK53_ARATH Probable WRKY transcription factor 53 |
| GSMUA_Achr9G12070_001 | 1.88 |  | 120.35 |  | 5.99 | sp|Q69VG1|CIGR1_ORYSJ Chitin-inducible gibberellin-responsive protein 1 |
| GSMUA_Achr8G03610_001 | 4.72 |  | 140.29 |  | 4.88 | sp|Q69VG1|CIGR1_ORYSJ Chitin-inducible gibberellin-responsive protein 1 |
| GSMUA_Achr9G26580_001 | 3.53 |  | 113.58 |  | 4.99 | sp|Q96289|ZAT10_ARATH Zinc finger protein ZAT10 |
| GSMUA_Achr6G32910_001 | 61.36 | 391.83 | 627.94 | 2.54 | 3.34 | sp|Q688R3|C3H33_ORYSJ Zinc finger CCCH domain-containing protein 33 |
| GSMUA_Achr8G25980_001 | 97.02 |  | 235.18 |  | 1.26 | sp|Q9FU27|C3H2_ORYSJ Zinc finger CCCH domain-containing protein 2 |
| GSMUA_Achr10G01300_001 | 7.21 |  | 110.64 |  | 3.93 | sp|Q9P1Y6|PHRF1_HUMAN PHD and RING finger domain-containing protein 1 |
| GSMUA_Achr9G14370_001 | 11.64 |  | 71.62 |  | 2.61 | sp|Q9S7M2|TI10B_ARATH Protein TIFY 10B |
| GSMUA_Achr8G21550_001 | 50.91 |  | 282.87 |  | 2.46 | sp|Q9SLH0|EIL1_ARATH ETHYLENE INSENSITIVE 3-like 1 protein |
| GSMUA_Achr9G17080_001 | 92.98 |  | 303.28 |  | 1.69 | sp|Q9SLH0|EIL1_ARATH ETHYLENE INSENSITIVE 3-like 1 protein |
| GSMUA_Achr11G05140_001 | 194.15 | 783.86 | 838.01 | 1.87 | 2.09 | sp|P24068|OCS1_MAIZE Ocs element-binding factor 1 |
| GSMUA_Achr7G11200_001 | 51.41 |  | 176.35 |  | 1.76 | sp|Q2R2W1|ADO3_ORYSJ Adagio-like protein 3 |
| GSMUA_Achr2G13670_001 | 128.67 |  | 403.73 |  | 1.63 | sp|O23310|NFYB3_ARATH Nuclear transcription factor Y subunit B-3 |
| GSMUA_Achr10G26880_001 | 398.30 | 173.09 | 120.11 | −1.35 | −1.74 | sp|Q8RWD0|COL16_ARATH Zinc finger protein CONSTANS-LIKE 16 |
| GSMUA_Achr4G30670_001 | 120.19 |  | 29.37 |  | −2.05 | sp|Q8RWD0|COL16_ARATH Zinc finger protein CONSTANS-LIKE 16 |
| GSMUA_Achr11G02890_001 | 779.71 | 270.05 | 190.63 | −1.64 | −2.05 | sp|A6MMN9|RPOA_DIOEL DNA-directed RNA polymerase subunit alpha |
| GSMUA_Achr3G04360_001 | 110.73 |  | 2.82 |  | −5.31 | sp|Q9LM15|RA213_ARATH Ethylene-responsive transcription factor RAP2-13 |
| Response to stress |  |  |  |  |  |  |
| GSMUA_Achr8G11980_001 | 0.00 |  | 28.64 |  | Inf | sp|Q6EP77|DRE1G_ORYSJ Dehydration-responsive element-binding protein 1G |
| GSMUA_Achr7G05900_001 | 5.06 | 375.22 | 1227.60 | 6.09 | 7.91 | sp|Q9LWV3|DRE1D_ORYSJ Dehydration-responsive element-binding protein 1D |
| GSMUA_Achr6G14750_001 | 0.00 |  | 35.49 |  | Inf | sp|Q9SYM2|STHY_ARATH Probable salt tolerance-like protein At1g78600 |
| GSMUA_Achr5G07340_001 | 0.68 |  | 108.90 |  | 7.31 | sp|Q10MX1|P2C32_ORYSJ Probable protein phosphatase 2C 32 |
| GSMUA_Achr9G20550_001 | 3.97 |  | 299.40 |  | 6.22 | sp|Q9SMT7|4CLLA_ARATH 4-coumarate--CoA ligase-like 10 |
| GSMUA_Achr9G03090_001 | 0.46 |  | 21.98 |  | 5.56 | sp|Q9C7A2|Y3236_ARATH Ankyrin repeat-containing protein At3g12360 |
| GSMUA_Achr2G13410_001 | 7.76 | 108.91 | 301.02 | 3.69 | 5.26 | sp|Q42430|ZFP1_WHEAT Zinc finger protein 1 |
| GSMUA_Achr1G08716_001 | 4.11 |  | 126.89 |  | 4.93 | sp|Q84WD3|DRL26_ARATH Probable disease resistance protein At4g19060 |
| GSMUA_Achr6G21800_001 | 4.14 |  | 36.70 |  | 3.14 | sp|Q7XA40|RGA3_SOLBU Putative disease resistance protein RGA3 |
| GSMUA_Achr9G17060_001 | 2.51 |  | 19.44 |  | 2.94 | sp|Q7XA40|RGA3_SOLBU Putative disease resistance protein RGA3 |
| GSMUA_Achr4G28700_001 | 26.68 |  | 75.27 |  | 1.48 | sp|O64973|RPS5_ARATH Disease resistance protein RPS5 |
| GSMUA_Achr6G13020_001 | 20.88 | 169.78 | 604.09 | 2.88 | 4.84 | sp|Q8LGD5|MKS1_ARATH Protein MKS1 |
| GSMUA_Achr9G26190_001 | 4.00 |  | 83.65 |  | 4.36 | sp|P81370|TLP_ACTDE Thaumatin-like protein |
| GSMUA_Achr5G08730_001 | 23.84 |  | 458.15 |  | 4.25 | sp|O22533|ZAT6_ARATH Zinc finger protein ZAT6 |
| GSMUA_Achr3G11070_001 | 148.93 | 597.55 | 2592.58 | 1.87 | 4.11 | sp|Q84PD8|SAP11_ORYSJ Zinc finger A20 and AN1 domain-containing stress-associated protein 11 |
| GSMUA_Achr9G19860_001 | 20.68 |  | 354.06 |  | 4.08 | sp|Q84PD8|SAP11_ORYSJ Zinc finger A20 and AN1 domain-containing stress-associated protein 11 |
| GSMUA_Achr10G22580_001 | 20.49 | 107.41 | 333.41 | 2.25 | 4.01 | sp|P0CH30|RING1_GOSHI E3 ubiquitin-protein ligase RING1 |
| GSMUA_Achr10G27000_001 | 14.89 | 95.82 | 198.82 | 2.55 | 3.72 | sp|Q69XJ0|SPX1_ORYSJ SPX domain-containing protein 1 |
| GSMUA_Achr6G33100_001 | 4.19 |  | 52.77 |  | 3.64 | sp|Q10N20|MPK5_ORYSJ Mitogen-activated protein kinase 5 |
| GSMUA_Achr7G10150_001 | 11.00 |  | 123.76 |  | 3.48 | sp|Q69XJ0|SPX1_ORYSJ SPX domain-containing protein 1 |
| GSMUA_Achr1G00680_001 | 13.82 |  | 103.59 |  | 2.89 | sp|Q9SUS5|RHA1B_ARATH E3 ubiquitin-protein ligase RHA1B |
| GSMUA_Achr4G30690_001 | 38.58 |  | 284.50 |  | 2.87 | sp|B8B4D0|SPX1_ORYSI SPX domain-containing protein 1 |
| GSMUA_Achr6G03290_001 | 4.59 |  | 32.44 |  | 2.81 | sp|P07788|COTA_BACSU Spore coat protein A |
| GSMUA_Achr6G15760_001 | 78.41 |  | 526.50 |  | 2.73 | sp|P20144|WUN1_SOLTU Wound-induced protein 1 |
| GSMUA_Achr7G00180_001 | 262.66 | 942.80 | 1680.04 | 1.70 | 2.66 | sp|F6H7K5|THI42_VITVI Thiamine thiazole synthase 2, chloroplastic |
| GSMUA_Achr3G26020_001 | 146.62 |  | 627.71 |  | 2.08 | sp|P37707|B2_DAUCA B2 protein |
| GSMUA_Achr5G07190_001 | 14.26 |  | 57.82 |  | 2.00 | sp|Q9C7A2|Y3236_ARATH Ankyrin repeat-containing protein At3g12360 |
| GSMUA_Achr4G32370_001 | 19.34 |  | 68.15 |  | 1.80 | sp|Q7Y0C8|C74A1_ORYSJ Allene oxide synthase 1, chloroplastic |
| GSMUA_Achr9G25940_001 | 27.48 |  | 93.73 |  | 1.76 | sp|Q04960|DNJH_CUCSA DnaJ protein homolog |
| GSMUA_Achr1G11430_001 | 119.37 |  | 344.42 |  | 1.52 | sp|Q94AR4|CID2_ARATH Polyadenylate-binding protein-interacting protein 2 |
| GSMUA_Achr9G11620_001 | 242.43 |  | 603.17 |  | 1.30 | sp|O49710|HS154_ARATH 15.4 kDa class V heat shock protein |
| GSMUA_Achr7G27650_001 | 423.47 |  | 1014.11 |  | 1.25 | sp|P49310|GRP1_SINAL Glycine-rich RNA-binding protein GRP1A |
| GSMUA_Achr4G11100_001 | 1409.86 |  | 606.31 |  | −1.23 | sp|Q39195|PST2_ARATH Photosystem II 5 kDa protein, chloroplastic |
| GSMUA_Achr5G09420_001 | 1031.06 |  | 417.05 |  | −1.32 | sp|Q942D4|BURP3_ORYSJ BURP domain-containing protein 3 |
| GSMUA_Achr5G15820_001 | 251.07 |  | 99.24 |  | −1.35 | sp|P30567|CATA2_GOSHI Catalase isozyme 2 |
| GSMUA_Achr6G10590_001 | 108.01 |  | 32.20 |  | −1.76 | sp|Q9LV93|AB5F_ARATH ABC transporter F family member 5 |
| GSMUA_Achr3G03820_001 | 312.35 |  | 81.11 |  | −1.96 | sp|Q00874|DR100_ARATH DNA-damage-repair/toleration protein DRT100 |
| Transport |  |  |  |  |  |  |
| GSMUA_Achr9G29680_001 | 2.25 |  | 143.35 |  | 5.98 | sp|Q9C615|SYP24_ARATH Putative syntaxin-24 |
| GSMUA_Achr5G11970_001 | 5.44 |  | 58.06 |  | 3.40 | sp|Q9C615|SYP24_ARATH Putative syntaxin-24 |
| GSMUA_Achr8G03020_001 | 10.00 |  | 88.45 |  | 3.13 | sp|Q9ZSD4|SY121_ARATH Syntaxin-121 |
| GSMUA_Achr6G31140_001 | 22.00 |  | 153.43 |  | 2.78 | sp|Q9ZSD4|SY121_ARATH Syntaxin-121 |
| GSMUA_Achr4G30280_001 | 3.39 |  | 110.65 |  | 5.02 | sp|Q8H074|PT112_ORYSJ Probable inorganic phosphate transporter 1-12 |
| GSMUA_Achr10G25070_001 | 93.77 | 516.39 | 889.02 | 2.31 | 3.23 | sp|Q9CR62|M2OM_MOUSE Mitochondrial 2-oxoglutarate/malate carrier protein |
| GSMUA_Achr7G22310_001 | 11.14 |  | 104.48 |  | 3.21 | sp|O80915|PR1B4_ARATH PRA1 family protein B4 |
| GSMUA_Achr8G32370_001 | 39.94 |  | 186.37 |  | 2.21 | sp|Q84WF5|VAMPL_ARATH Probable VAMP-like protein At1g33475 |
| GSMUA_Achr9G01100_001 | 16.10 |  | 68.38 |  | 2.07 | sp|Q9LYR6|PTR49_ARATH Probable peptide/nitrate transporter At5g13400 |
| GSMUA_Achr4G26750_001 | 25.58 |  | 90.82 |  | 1.81 | sp|Q8H6H2|PHT14_ORYSJ Probable inorganic phosphate transporter 1-4 |
| GSMUA_Achr1G22700_001 | 454.08 |  | 1368.00 |  | 1.58 | sp|Q8VZ80|PLT5_ARATH Polyol transporter 5 |
| GSMUA_Achr4G16700_001 | 58.75 |  | 172.89 |  | 1.54 | sp|Q39950|NLTP_HELAN Non-specific lipid-transfer protein |
| GSMUA_Achr9G09130_001 | 230.09 |  | 111.67 |  | −1.06 | sp|P73627|Y1770_SYNY3 Uncharacterized protein sll1770 |
| GSMUA_Achr4G10972_001 | 11197.01 |  | 3750.99 |  | −1.59 | sp|A6MML9|CEMA_DIOEL Chloroplast envelope membrane protein |
| GSMUA_Achr11G00890_001 | 620.29 |  | 179.82 |  | −1.80 | sp|Q8R1R3|STAR7_MOUSE StAR-related lipid transfer protein 7, mitochondrial |
| GSMUA_Achr2G07910_001 | 999.95 |  | 124.25 |  | −3.03 | sp|Q9SZN7|HIP26_ARATH Heavy metal-associated isoprenylated plant protein 26 |
| Protein modification |  |  |  |  |  |  |
| GSMUA_Achr7G22130_001 | 0.42 | 26.38 | 225.08 | 5.82 | 9.06 | sp|Q9LZW3|PUB16_ARATH U-box domain-containing protein 16 |
| GSMUA_Achr4G00200_001 | 10.37 |  | 130.20 |  | 3.64 | sp|Q9LZW3|PUB16_ARATH U-box domain-containing protein 16 |
| GSMUA_Achr6G25670_001 | 103.79 | 318.57 | 1264.79 | 1.49 | 3.59 | sp|Q9LT79|PUB25_ARATH U-box domain-containing protein 25 |
| GSMUA_Achr9G08750_001 | 19.84 |  | 239.09 |  | 3.58 | sp|Q9LT79|PUB25_ARATH U-box domain-containing protein 25 |
| GSMUA_Achr9G28820_001 | 5.27 |  | 62.64 |  | 3.56 | sp|Q9C7R6|PUB17_ARATH U-box domain-containing protein 17 |
| GSMUA_Achr7G26200_001 | 7.83 |  | 90.67 |  | 3.52 | sp|O22193|PUB4_ARATH U-box domain-containing protein 4 |
| GSMUA_Achr8G04940_001 | 5.98 |  | 43.02 |  | 2.83 | sp|Q058P4|PUB30_ARATH U-box domain-containing protein 30 |
| GSMUA_Achr9G07530_001 | 13.40 |  | 193.15 |  | 3.84 | sp|Q02527|MGAT3_RAT Beta-1,4-mannosyl-glycoprotein 4-beta-N-acetylglucosaminyltransferase |
| GSMUA_Achr6G28040_001 | 24.98 |  | 83.36 |  | 1.72 | sp|Q02527|MGAT3_RAT Beta-1,4-mannosyl-glycoprotein 4-beta-N-acetylglucosaminyltransferase |
| GSMUA_Achr2G13700_001 | 11.41 |  | 81.81 |  | 2.83 | sp|Q10469|MGAT2_HUMAN Alpha-1,6-mannosyl-glycoprotein 2-beta-N-acetylglucosaminyltransferase |
| GSMUA_Achr4G07730_001 | 5.98 |  | 68.43 |  | 3.50 | sp|Q9CY62|RN181_MOUSE E3 ubiquitin-protein ligase RNF181 |
| GSMUA_Achr2G17260_001 | 4.02 |  | 33.42 |  | 3.04 | sp|C0LGK4|Y2165_ARATH Probable LRR receptor-like serine/threonine-protein kinase At2g16250 |
| GSMUA_Achr1G23790_001 | 14.16 |  | 113.73 |  | 2.99 | sp|Q8GX29|SKI25_ARATH F-box/kelch-repeat protein SKIP25 |
| GSMUA_Achr11G02820_001 | 25.03 |  | 95.50 |  | 1.92 | sp|Q9FLW0|Y5241_ARATH Probable receptor-like protein kinase At5g24010 |
| GSMUA_Achr9G01670_001 | 147.37 |  | 52.13 |  | −1.52 | sp|Q9SRT0|PUB9_ARATH U-box domain-containing protein 9 |
| Nucleosome assembly |  |  |  |  |  |  |
| GSMUA_Achr5G03220_001 | 3.91 |  | 50.20 |  | 3.67 | sp|P27806|H1_WHEAT Histone H1 |
| GSMUA_Achr4G17770_001 | 12.53 |  | 76.09 |  | 2.59 | sp|P23444|H1_MAIZE Histone H1 |
| GSMUA_Achr7G12120_001 | 363.61 |  | 1282.88 |  | 1.80 | sp|Q9M5W4|H1_EUPES Histone H1 |
| GSMUA_Achr8G07360_001 | 15.64 |  | 116.35 |  | 2.88 | sp|Q6ZL42|H2A2_ORYSJ Probable histone H2A.2 |
| GSMUA_Achr10G10050_001 | 72.64 |  | 434.96 |  | 2.57 | sp|Q6ZL42|H2A2_ORYSJ Probable histone H2A.2 |
| GSMUA_Achr2G19080_001 | 42.78 |  | 212.39 |  | 2.30 | sp|Q943L2|H2B11_ORYSJ Histone H2B.11 |
| GSMUA_Achr6G33320_001 | 197.57 |  | 494.70 |  | 1.31 | sp|Q943L2|H2B11_ORYSJ Histone H2B.11 |
| GSMUA_Achr8G06850_001 | 31.37 |  | 225.31 |  | 2.83 | sp|Q9S9K7|H2AXB_ARATH Probable histone H2AXb |
| GSMUA_Achr1G26160_001 | 10.23 |  | 814.23 |  | 6.31 | sp|P68428|H32_WHEAT Histone H3.2 |
| GSMUA_Achr2G03260_001 | 137.97 |  | 365.83 |  | 1.39 | sp|Q71H73|H33_VITVI Histone H3.3 |
| GSMUA_Achr3G27970_001 | 49.57 |  | 299.36 |  | 2.58 | sp|P0CG89|H4_SOYBN Histone H4 |
| GSMUA_Achr1G07240_001 | 60.51 | 220.33 | 297.60 | 1.73 | 2.28 | sp|P0CG89|H4_SOYBN Histone H4 |
| Cell wall organization and biogenesis |  |  |  |  |  |  |
| GSMUA_Achr10G23480_001 | 0.00 |  | 54.19 |  | Inf | sp|Q38907|XTH25_ARATH Probable xyloglucan endotransglucosylase/hydrolase protein 25 |
| GSMUA_Achr3G05220_001 | 0.00 |  | 78.53 |  | Inf | sp|Q38910|XTH23_ARATH Probable xyloglucan endotransglucosylase/hydrolase protein 23 |
| GSMUA_Achr5G21880_001 | 0.00 | 39.48 | 139.05 | Inf | Inf | sp|Q38910|XTH23_ARATH Probable xyloglucan endotransglucosylase/hydrolase protein 23 |
| GSMUA_Achr9G13820_001 | 3.37 |  | 144.43 |  | 5.42 | sp|Q38910|XTH23_ARATH Probable xyloglucan endotransglucosylase/hydrolase protein 23 |
| GSMUA_Achr5G12160_001 | 0.00 |  | 43.12 |  | Inf | sp|Q0V7R1|GATL3_ARATH Probable galacturonosyltransferase-like 3 |
| GSMUA_Achr5G07510_001 | 0.44 |  | 24.92 |  | 5.79 | sp|P0C1U4|GUN9_ORYSJ Endoglucanase 9 |
| GSMUA_Achr7G21070_001 | 11.60 | 92.32 | 152.48 | 2.84 | 3.70 | sp|Q67VS7|CSLA9_ORYSJ Probable mannan synthase 9 |
| GSMUA_Achr7G08810_001 | 2.41 |  | 20.20 |  | 3.05 | sp|Q9LHF1|LRX4_ARATH Leucine-rich repeat extensin-like protein 4 |
| GSMUA_Achr11G01740_001 | 17.97 |  | 68.64 |  | 1.92 | sp|Q5PQ30|LYSM1_XENLA LysM and putative peptidoglycan-binding domain-containing protein 1 |
| GSMUA_Achr11G02340_001 | 148.22 | 45.31 |  | −1.83 |  | sp|Q9LXV0|U92A1_ARATH UDP-glycosyltransferase 92A1 |
| GSMUA_Achr11G19780_001 | 133.68 | 14.02 | 26.18 | −3.37 | −2.37 | sp|O22874|EXPA8_ARATH Expansin-A8 |
| GSMUA_Achr7G22100_001 | 217.91 | 15.33 | 29.66 | −4.02 | −2.90 | sp|Q0DHB7|EXPA4_ORYSJ Expansin-A4 |
| Signal transduction |  |  |  |  |  |  |
| GSMUA_Achr7G26970_001 | 3.01 |  | 85.58 |  | 4.82 | sp|Q6Z9F4|CIPK6_ORYSJ CBL-interacting protein kinase 6 |
| GSMUA_Achr6G14530_001 | 10.76 |  | 66.09 |  | 2.61 | sp|Q9LP51|CIPKI_ARATH CBL-interacting serine/threonine-protein kinase 18 |
| GSMUA_Achr9G04780_001 | 4.41 | 51.36 | 157.55 | 3.41 | 5.15 | sp|O22932|CIPKB_ARATH CBL-interacting serine/threonine-protein kinase 11 |
| GSMUA_Achr8G09680_001 | 125.54 | 373.01 |  | 1.44 |  | sp|Q9SS31|CML36_ARATH Probable calcium-binding protein CML36 |
| GSMUA_Achr3G23000_001 | 26.42 | 314.01 |  | 3.45 |  | sp|Q9ZPX9|KIC_ARATH Calcium-binding protein KIC |
| GSMUA_Achr1G03980_001 | 52.99 |  | 524.57 |  | 3.29 | sp|Q9ZPX9|KIC_ARATH Calcium-binding protein KIC |
| GSMUA_Achr5G26360_001 | 3.04 |  | 79.71 |  | 4.69 | sp|Q9ZPX9|KIC_ARATH Calcium-binding protein KIC |
| GSMUA_Achr3G16870_001 | 4.53 | 88.80 | 282.39 | 4.14 | 5.95 | sp|Q9SRR7|CML3_ARATH Calmodulin-like protein 3 |
| GSMUA_Achr9G07990_001 | 66.04 |  | 285.67 |  | 2.10 | sp|O22845|CML5_ARATH Calmodulin-like protein 5 |
| Oxidation reduction |  |  |  |  |  |  |
| GSMUA_Achr6G07480_001 | 0.88 |  | 91.22 |  | 6.68 | sp|P85191|CP450_HELAN Cytochrome P450 (Fragment) |
| GSMUA_Achr2G08560_001 | 15.33 |  | 147.52 |  | 3.25 | sp|O81117|C94A1_VICSA Cytochrome P450 94A1 |
| GSMUA_Achr7G26580_001 | 34.34 | 266.97 | 1106.93 | 2.81 | 4.99 | sp|Q96558|UGDH_SOYBN UDP-glucose 6-dehydrogenase |
| GSMUA_Achr7G10990_001 | 6.65 |  | 94.00 |  | 3.81 | sp|Q0JP62|GRXS3_ORYSJ Monothiol glutaredoxin-S3 |
| GSMUA_Achr2G15240_001 | 81.70 | 306.09 | 1052.99 | 1.77 | 3.67 | sp|P0C291|GRXS9_ORYSJ Monothiol glutaredoxin-S9 |
| GSMUA_Achr6G23230_001 | 3.33 |  | 41.96 |  | 3.64 | sp|Q08632|SDR1_PICAB Short-chain type dehydrogenase/reductase |
| GSMUA_Achr5G29460_001 | 254.00 | 64.82 |  | −2.10 |  | sp|Q948P6|FRI3_SOYBN Ferritin-3, chloroplastic |
| RNA modification |  |  |  |  |  |  |
| GSMUA_Achr9G29700_001 | 15.04 | 140.86 | 333.84 | 3.09 | 4.46 | sp|Q9FMS6|CAF1K_ARATH Probable CCR4-associated factor 1 homolog 11 |
| GSMUA_Achr6G19450_001 | 2.90 |  | 59.95 |  | 4.35 | sp|Q9FMS6|CAF1K_ARATH Probable CCR4-associated factor 1 homolog 11 |
| GSMUA_Achr2G05120_001 | 35.78 | 167.91 | 431.07 | 2.10 | 3.58 | sp|Q9SKZ2|CAF1G_ARATH Probable CCR4-associated factor 1 homolog 7 |
| GSMUA_Achr4G11450_001 | 57.92 |  | 386.40 |  | 2.72 | sp|Q9SKZ2|CAF1G_ARATH Probable CCR4-associated factor 1 homolog 7 |
| Cell redox homeostasis |  |  |  |  |  |  |
| GSMUA_Achr3G25160_001 | 0.55 |  | 22.31 |  | 5.35 | sp|Q84JR9|TTL4_ARATH TPR repeat-containing thioredoxin TTL4 |
| GSMUA_Achr8G22080_001 | 122.96 |  | 288.75 |  | 1.22 | sp|Q9FLE8|Y5986_ARATH Uncharacterized protein At5g39865 |
| GSMUA_Achr10G15090_001 | 1689.81 |  | 604.94 |  | −1.50 | sp|Q9FLE8|Y5986_ARATH Uncharacterized protein At5g39865 |
| Translation |  |  |  |  |  |  |
| GSMUA_Achr11G15530_001 | 14518.49 |  | 4449.90 |  | −1.72 | sp|Q3BAJ7|RK22_PHAAO 50S ribosomal protein L22, chloroplastic |
| GSMUA_Achr6G06682_001 | 1183.11 |  | 344.83 |  | −1.79 | sp|Q3V4X7|RR15_ACOCL 30S ribosomal protein S15, chloroplastic |
| GSMUA_Achr3G31260_001 | 6673.16 |  | 1827.12 |  | −1.88 | sp|Q3V4Z3|RR19_ACOCL 30S ribosomal protein S19, chloroplastic |
| Lipid metabolism |  |  |  |  |  |  |
| GSMUA_Achr6G36960_001 | 5.15 |  | 155.48 |  | 4.90 | sp|O23522|PLA14_ARATH Phospholipase A1-Ibeta2, chloroplastic |
| GSMUA_Achr5G21240_001 | 1.12 |  | 29.47 |  | 4.69 | sp|P19515|LIP_RHIMI Lipase |
| Nucleic acid phosphodiester bond hydrolysis |  |  |  |  |  |  |
| GSMUA_Achr5G22180_001 | 6.74 |  | 841.52 |  | 6.95 | sp|Q17QR8|HARB1_BOVIN Putative nuclease HARBI1 |
| GSMUA_Achr7G24010_001 | 0.74 |  | 84.75 |  | 6.83 | sp|Q17QR8|HARB1_BOVIN Putative nuclease HARBI1 |
| Phospholipid biosynthesis |  |  |  |  |  |  |
| GSMUA_Achr5G27160_001 | 11.73 |  | 0.00 |  | -Inf | sp|Q9SYJ2|GPAT3_ARATH Probable glycerol-3-phosphate acyltransferase 3 |
| GSMUA_Achr8G27070_001 | 40.78 |  | 7.34 |  | −2.49 | sp|Q9SHJ5|GPAT1_ARATH Glycerol-3-phosphate acyltransferase 1 |
| S-adenosylmethionine biosynthesis |  |  |  |  |  |  |
| GSMUA_Achr3G08470_001 | 23.91 |  | 120.30 |  | 2.32 | sp|Q944U4|METK_DENCR S-adenosylmethionine synthase |
| GSMUA_Achr8G06200_001 | 585.24 |  | 1394.85 |  | 1.24 | sp|O22338|METK_MUSAC S-adenosylmethionine synthase |
| Ubiquitin-dependent protein catabolic process |  |  |  |  |  |  |
| GSMUA_Achr6G18950_001 | 104.16 |  | 501.85 |  | 2.25 | sp|P19848|UBIQ_COPCO Ubiquitin |
| GSMUA_Achr6G18960_001 | 286.16 |  | 1352.07 |  | 2.23 | sp|P0CH33|UBQ11_ARATH Polyubiquitin 11 |
| Ethylene biosynthesis |  |  |  |  |  |  |
| GSMUA_Achr6G12910_001 | 0.00 |  | 32.65 |  | Inf | sp|Q9FR99|ACCO_MUSAC 1-aminocyclopropane-1-carboxylate oxidase |
| Iron-sulfur cluster assembly |  |  |  |  |  |  |
| GSMUA_Achr9G07610_001 | 0.00 | 48.29 | 266.18 | Inf | Inf | sp|Q6FJ73|CIAO1_CANGA Probable cytosolic iron-sulfur protein assembly protein 1 |
| Phenylalanine biosynthesis |  |  |  |  |  |  |
| GSMUA_Achr6G17230_001 | 2.67 |  | 92.80 |  | 5.10 | sp|Q9ZUY3|AROD3_ARATH Arogenate dehydratase 3, chloroplastic |
| Proteolysis |  |  |  |  |  |  |
| GSMUA_Achr11G04410_001 | 3.26 |  | 77.06 |  | 4.54 | sp|Q766C3|NEP1_NEPGR Aspartic proteinase nepenthesin-1 |
| Epidermal cell fate specification |  |  |  |  |  |  |
| GSMUA_Achr4G29160_001 | 4.59 |  | 68.86 |  | 3.89 | sp|Q9XGN1|TTG1_ARATH Protein TRANSPARENT TESTA GLABRA 1 |
| Fatty acid biosynthesis |  |  |  |  |  |  |
| GSMUA_Achr7G08410_001 | 7.27 |  | 94.10 |  | 3.68 | sp|O48780|KCS11_ARATH 3-ketoacyl-CoA synthase 11 |
| Cellulose microfibril organization |  |  |  |  |  |  |
| GSMUA_Achr11G15850_001 | 3.45 |  | 32.58 |  | 3.23 | sp|Q8GZ17|COBL7_ARATH COBRA-like protein 7 |
| Gibberellin signaling pathway |  |  |  |  |  |  |
| GSMUA_Achr8G05900_001 | 6.33 |  | 56.06 |  | 3.13 | sp|Q940G6|GID1C_ARATH Gibberellin receptor GID1C |
| Regulation of endopeptidase activity |  |  |  |  |  |  |
| GSMUA_Achr1G05200_001 | 145.08 |  | 403.31 |  | 1.46 | sp|P80321|IBB_MEDSC Bowman-Birk type proteinase inhibitor |
| Intracellular distribution of mitochondria |  |  |  |  |  |  |
| GSMUA_Achr5G04650_001 | 66.01 |  | 27.13 |  | −1.30 | sp|O15818|K0664_DICDI Protein KIAA0664 homolog |
| Regulation of protein import into nucleus |  |  |  |  |  |  |
| GSMUA_Achr7G24630_001 | 145.70 |  | 57.63 |  | −1.35 | sp|Q54G05|LRRX1_DICDI Putative leucine-rich repeat-containing protein DDB_G0290503 |
| Photosynthesis |  |  |  |  |  |  |
| GSMUA_Achr6G36680_001 | 1254.53 |  | 495.85 |  | −1.35 | sp|Q09FZ0|NDHH_PLAOC NAD(P)H-quinone oxidoreductase subunit H, chloroplastic |
| Starch biosynthesis |  |  |  |  |  |  |
| GSMUA_Achr1G06140_001 | 183.17 |  | 66.87 |  | −1.47 | sp|O82627|SSG1_ANTMA Granule-bound starch synthase 1, chloroplastic/amyloplastic |
| Protein folding |  |  |  |  |  |  |
| GSMUA_Achr1G10150_001 | 613.86 |  | 207.66 |  | −1.58 | sp|Q9SAG8|DNAJ8_ARATH Chaperone protein dnaJ 8, chloroplastic |
| Chromatin modification |  |  |  |  |  |  |
| GSMUA_Achr4G23560_001 | 128.03 |  | 37.80 |  | −1.77 | sp|Q7TNV0|DEK_MOUSE Protein DEK |
| Chloroplast accumulation movement |  |  |  |  |  |  |
| GSMUA_Achr1G23190_001 | 92.54 |  | 24.96 |  | −1.90 | sp|Q9C9N6|PMI2_ARATH Protein PLASTID MOVEMENT IMPAIRED 2 |
| Response to auxin stimulus |  |  |  |  |  |  |
| GSMUA_Achr4G21030_001 | 361.51 |  | 52.57 |  | −2.80 | sp|O24542|AX22D_VIGRR Auxin-induced protein 22D |
| Glucosinolate biosynthesis |  |  |  |  |  |  |
| GSMUA_Achr4G14500_001 | 106.35 |  | 11.52 |  | −3.22 | sp|Q9C9C9|SOT18_ARATH Cytosolic sulfotransferase 18 |
| Unknown |  |  |  |  |  |  |
| GSMUA_Achr5G06470_001 | 0.00 |  | 11.17 |  | Inf | Putative uncharacterized protein |
| GSMUA_Achr10G24630_001 | 0.00 |  | 139.88 |  | Inf | Putative uncharacterized protein |
| GSMUA_Achr10G22560_001 | 0.00 |  | 72.13 |  | Inf | Putative uncharacterized protein |
| GSMUA_Achr3G29120_001 | 0.00 |  | 98.38 |  | Inf | Putative uncharacterized protein |
| GSMUA_Achr6G09160_001 | 0.00 |  | 34.92 |  | Inf | Putative uncharacterized protein |
| GSMUA_Achr6G14410_001 | 0.00 |  | 138.82 |  | Inf | Putative uncharacterized protein |
| GSMUA_Achr10G28690_001 | 0.00 |  | 29.03 |  | Inf | Putative uncharacterized protein |
| GSMUA_Achr10G24450_001 | 0.00 |  | 181.25 |  | Inf | Putative uncharacterized protein |
| GSMUA_Achr4G20610_001 | 0.00 |  | 70.92 |  | Inf | Putative uncharacterized protein |
| GSMUA_Achr11G04930_001 | 0.00 |  | 103.84 |  | Inf | Putative uncharacterized protein |
| GSMUA_Achr9G13120_001 | 4.60 |  | 123.57 |  | 4.73 | Putative uncharacterized protein |
| GSMUA_Achr1G08710_001 | 1.86 |  | 49.51 |  | 4.72 | Putative uncharacterized protein |
| GSMUA_Achr2G17220_001 | 2.96 |  | 76.15 |  | 4.66 | Putative uncharacterized protein |
| GSMUA_Achr6G01040_001 | 2.94 |  | 68.23 |  | 4.52 | Putative uncharacterized protein |
| GSMUA_Achr1G24700_001 | 7.69 |  | 176.49 |  | 4.51 | Putative uncharacterized protein |
| GSMUA_Achr6G32080_001 | 7.48 |  | 160.80 |  | 4.41 | Putative uncharacterized protein |
| GSMUA_Achr9G12270_001 | 14.01 |  | 239.33 |  | 4.08 | Putative uncharacterized protein |
| GSMUA_Achr11G18110_001 | 20.56 |  | 299.20 |  | 3.85 | Putative uncharacterized protein |
| GSMUA_Achr9G21690_001 | 65.88 | 295.27 | 951.01 | 2.01 | 3.84 | Putative uncharacterized protein |
| GSMUA_Achr5G01760_001 | 44.49 |  | 502.55 |  | 3.48 | Putative uncharacterized protein |
| GSMUA_Achr7G13610_001 | 6.69 |  | 67.81 |  | 3.33 | sp|O65451|FB333_ARATH Probable F-box protein At4g22030 |
| GSMUA_Achr2G22290_001 | 21.59 |  | 217.85 |  | 3.32 | Putative uncharacterized protein |
| GSMUA_Achr6G10300_001 | 34.60 |  | 322.05 |  | 3.21 | Putative uncharacterized protein |
| GSMUA_Achr10G16780_001 | 2.88 |  | 25.20 |  | 3.11 | sp|Q84TF5|RHA4A_ARATH RING-H2 zinc finger protein RHA4a |
| GSMUA_Achr2G02570_001 | 27.35 |  | 227.64 |  | 3.04 | Putative uncharacterized protein |
| GSMUA_Achr9G30820_001 | 6.73 |  | 55.82 |  | 3.03 | Putative uncharacterized protein |
| GSMUA_Achr11G20670_001 | 37.51 |  | 309.52 |  | 3.03 | Putative uncharacterized protein |
| GSMUA_Achr2G22400_001 | 9.92 |  | 75.87 |  | 2.92 | Putative uncharacterized protein |
| GSMUA_Achr5G01410_001 | 10.52 |  | 75.83 |  | 2.84 | Putative uncharacterized protein |
| GSMUA_Achr5G11190_001 | 33.11 |  | 218.73 |  | 2.71 | Putative uncharacterized protein |
| GSMUA_Achr6G32740_001 | 8.90 |  | 56.26 |  | 2.65 | Putative uncharacterized protein |
| GSMUA_Achr7G12260_001 | 9.16 |  | 53.14 |  | 2.52 | Putative uncharacterized protein |
| GSMUA_Achr10G19940_001 | 33.50 |  | 180.86 |  | 2.42 | Putative uncharacterized protein |
| GSMUA_Achr1G20910_001 | 73.21 |  | 345.44 |  | 2.22 | Putative uncharacterized protein |
| GSMUA_Achr8G09020_001 | 64.08 |  | 246.43 |  | 1.93 | Putative uncharacterized protein |
| GSMUA_Achr10G30690_001 | 31.81 |  | 116.36 |  | 1.86 | Putative uncharacterized protein |
| GSMUA_Achr6G16830_001 | 68.84 |  | 247.81 |  | 1.83 | sp|Q9LYZ9|PP362_ARATH Pentatricopeptide repeat-containing protein At5g02860 |
| GSMUA_Achr2G13780_001 | 18.64 |  | 63.28 |  | 1.75 | sp|Q8LF09|FDL23_ARATH F-box/FBD/LRR-repeat protein At4g00160 |
| GSMUA_Achr7G01430_001 | 60.30 |  | 204.75 |  | 1.75 | sp|P05332|YP20_BACLI Uncharacterized N-acetyltransferase p20 |
| GSMUA_Achr1G01000_001 | 153.67 |  | 422.08 |  | 1.44 | Putative uncharacterized protein |
| GSMUA_Achr6G35120_001 | 768.40 |  | 1825.57 |  | 1.23 | Putative uncharacterized protein |
| GSMUA_Achr10G04290_001 | 461.48 |  | 166.80 |  | −1.48 | Putative uncharacterized protein |
| GSMUA_Achr2G06930_001 | 442.48 |  | 159.52 |  | −1.49 | sp|Q5HZL9|HDHD3_XENLA Haloacid dehalogenase-like hydrolase domain-containing protein 3 |
| GSMUA_Achr3G16560_001 | 62.90 |  | 22.51 |  | −1.50 | sp|Q9NUJ3|T11L1_HUMAN T-complex protein 11-like protein 1 |
| GSMUA_Achr9G20310_001 | 203.72 |  | 65.22 |  | −1.66 | Putative uncharacterized protein |
| GSMUA_Achr11G09090_001 | 175.81 |  | 53.83 |  | −1.72 | Putative uncharacterized protein |
| GSMUA_Achr7G13960_001 | 396.42 |  | 107.30 |  | −1.90 | Putative uncharacterized protein |
| GSMUA_Achr3G06040_001 | 198.16 |  | 50.72 |  | −1.98 | Putative uncharacterized protein |
| GSMUA_Achr2G21310_001 | 424.14 |  | 106.90 |  | −2.00 | Putative uncharacterized protein |
| GSMUA_Achr2G14250_001 | 107.16 |  | 25.89 |  | −2.06 | Putative uncharacterized protein |
| GSMUA_Achr3G25420_001 | 60.70 |  | 13.44 |  | −2.19 | sp|Q9FI40|BAHD1_ARATH BAHD acyltransferase At5g47980 |
| GSMUA_Achr8G30530_001 | 125.03 | 28.12 | 20.02 | −2.33 | −2.66 | Putative uncharacterized protein |
| GSMUA_Achr9G01390_001 | 104.79 |  | 9.17 |  | −3.53 | Putative uncharacterized protein |
| GSMUA_Achr10G24780_001 | 15.81 | 257.17 |  | 3.89 |  | Putative uncharacterized protein |
