# Supplementary figures and images for: Comparative transcriptomics analysis reveals difference of key gene expression between banana and plantain in response to cold stress
Source: BMC Genomics. 2015 Jun 10;16(1):446. doi: 10.1186/s12864-015-1551-z (PMC4461995; doi:10.1186/s12864-015-1551-z)

### E3 SUMO-protein ligase SIZ1

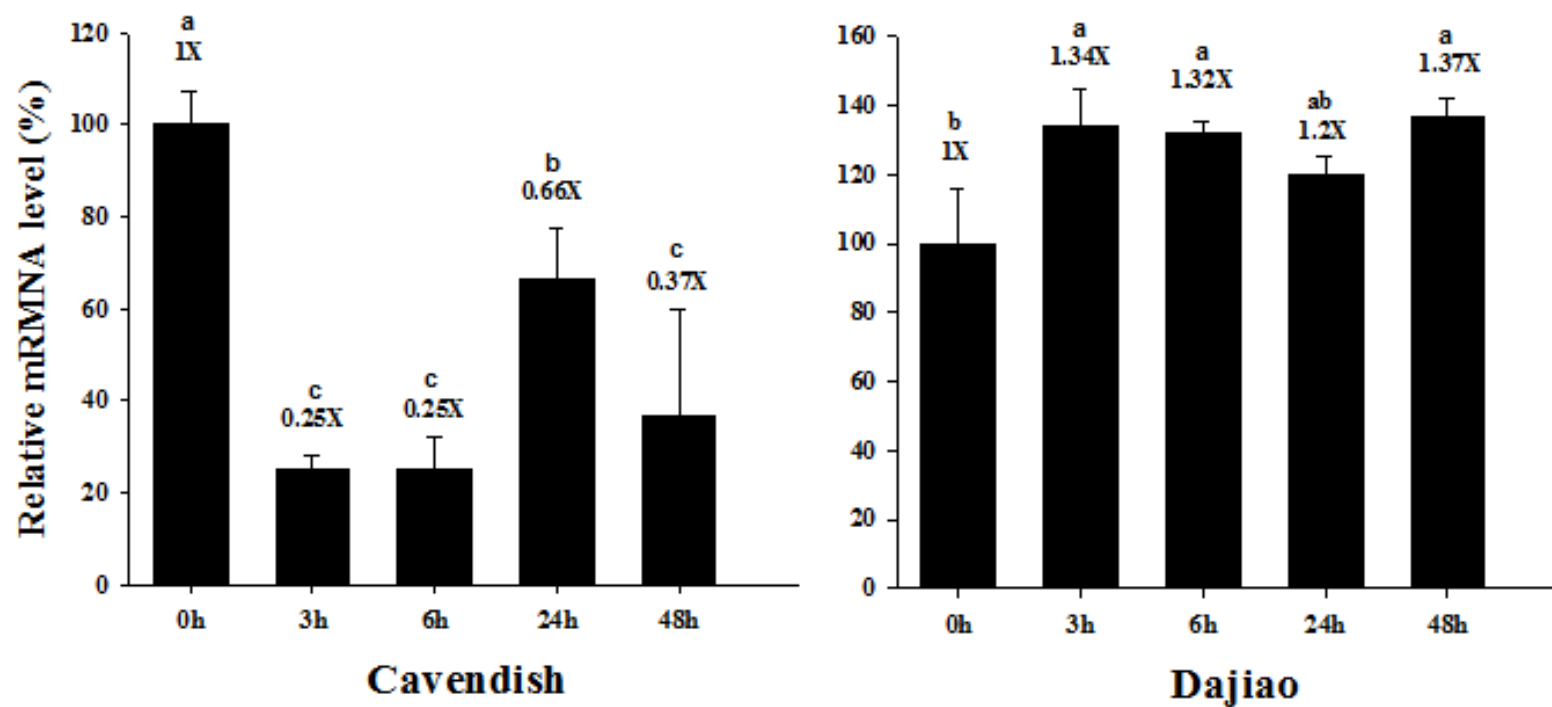

Fig S1

Supplement: Additional file 6: Figure S1. — Relative mRNA levels of E2 SUMO-protein ligase SIZ1 in banana and plantain seedlings were determined by quantitative RT-PCR analyses. Six-leaf stage seedlings were incubated at 10°C for the indicated time. Transcript abundances of genes encoding E2 SUMO-protein ligase SIZ1 from both banana and plantain were determined and compared across the time course of cold stress. Data represent means ± SD (n = 4). The different lowercase letters labeled above columns indicate a significant difference at p ≤ 0.05 between the columns by Duncan’s test using SPSS statistical software (version 16.0, SPSS Inc. Chicago, IL). The columns with the same letters mean no significant difference (p > 0.05) between each other. [file 12864_2015_1551_MOESM6_ESM.pdf]
